# Multivariate genetic analysis of plant responses to water deficit and high temperature revealed contrasting adaptive strategies

**DOI:** 10.1093/jxb/eru364

**Published:** 2014-09-22

**Authors:** François Vasseur, Thibaut Bontpart, Myriam Dauzat, Christine Granier, Denis Vile

**Affiliations:** INRA, Montpellier SupAgro, UMR759 Laboratoire d’Ecophysiologie des Plantes sous Stress Environnementaux (LEPSE), F-34060 Montpellier, France

**Keywords:** Antagonistic pleiotropy, *Arabidopsis thaliana*, genotype by environment interactions, **G**-matrix, mixed-effects model, photosynthesis, QTL, water use efficiency.

## Abstract

How genetic factors control plant performance under stressful environmental conditions is a central question in ecology and for crop breeding. A multivariate framework was developed to examine the genetic architecture of performance-related traits in response to interacting environmental stresses. Ecophysiological and life history traits were quantified in the *Arabidopsis thaliana* L*er*×Cvi mapping population exposed to constant soil water deficit and high air temperature. The plasticity of the genetic variance–covariance matrix (**G**-matrix) was examined using mixed-effects models after regression into principal components. Quantitative trait locus (QTL) analysis was performed on the predictors of genotype effects and genotype by environment interactions (G×E). Three QTLs previously identified for flowering time had antagonistic G×E effects on carbon acquisition and the other traits (phenology, growth, leaf morphology, and transpiration). This resulted in a size-dependent response of water use efficiency (WUE) to high temperature but not soil water deficit, indicating that most of the plasticity of carbon acquisition and WUE to temperature is controlled by the loci that control variation of development, size, growth, and transpiration. A fourth QTL, *MSAT2.22*, controlled the response of carbon acquisition to specific combinations of watering and temperature irrespective of plant size and development, growth, and transpiration rate, which resulted in size-independent plasticity of WUE. These findings highlight how the strategies to optimize plant performance may differ in response to water deficit and high temperature (or their combination), and how different G×E effects could be targeted to improve plant tolerance to these stresses.

## Summary

Plants need to control transpiration and photosynthesis tightly in order to grow under drought and high temperature. Contrasting genetic-by-environment interactions are exploited by *Arabidopsis thaliana* to improve stress tolerance.

## Introduction

Because of resource limitation and biophysical constraints, plants cannot simultaneously optimize competing functions, which inevitably generates relationships and trade-offs between traits. Natural variation in the traits related to plant performance defines a multidimensional phenotypic space in which selection for the optimal trait combination, or strategy, is expected (Wagner and Zhang, 2011). The maintenance of natural variation is expected because different strategies can be advantageous or disadvantageous depending on the environment, and because different genotypes exhibit contrasting response strategies (i.e. multidimensional phenotypic responses) to a change in the environment. The latter illustrates the presence of genotype by environment interactions (G×E) in the control of plant performance. However, it does not imply that there are G×E for all the traits that contribute to the processes underlying plant performance. The picture is even more complex since multiple environmental factors can differentially impact performance-related traits. Multivariate modelling approaches are thus needed to decipher the phenotypic and genotypic components of the integrated responses of plants to multiple environmental conditions.

Water deficit (WD) and high temperature (HT) are among the major stresses impairing plant growth and productivity in natural and field conditions (Boyer, 1982; Ciais *et al.*, 2005). The regulation of water and carbon fluxes plays a prominent role in the strategies to adapt to these stresses. For instance, water conservation through stomatal closure is important for resistance to drought but it penalizes leaf cooling through transpiration (Crawford *et al.*, 2012). In contrast, heat accelerates metabolic processes and energy consumption, but drought-induced stomatal closure reduces carbon fixation and sugar production (Vasseur *et al.*, 2011). The identification of genomic regions associated with a higher rate of carbon assimilation per unit of water consumed [i.e. a higher water use efficiency (WUE)] is thus a promising avenue for improving crop tolerance to both WD and HT. These two stresses often occur simultaneously in the field, but very few studies have investigated their combined effects on the genetic and phenotypic components of plant adaptation (Mittler, 2006). It was recently shown that the effects of HT and WD were additive for the traits related to biomass allocation across a set of *Arabidopsis thaliana* natural accessions (Vile *et al.*, 2012). However, the phenotypic responses differed greatly among accessions, which resulted from strong G×E effects for most of the studied traits.

The diversity of plant strategies results from standing genetic variation at loci that control the genetic variance–covariance matrix (**G**-matrix) between phenotypic traits. The structure and plasticity of the **G**-matrix are key determinants of the evolutionary adaptation of plant populations to abiotic stresses (Juenger, 2013). Hence, a common locus controlling multiple traits, termed a pleiotropic locus, leads to phenotypic correlations that constrain the independent variation of traits, and thus their response to selection. For instance, it was demonstrated that selection is facilitated in the direction of major axes of phenotypic variation and prevented in the other directions (Schluter, 1996). Fisher (1930) was the first to model the genetic structure of the phenotypic space by asserting that ‘every gene affects every trait’. However, with the development of molecular biology and quantitative genetics, Fisher’s view of universal pleiotropy was revisited (e.g. Martin and Lenormand, 2006; Pavlicev and Wagner, 2012). Recently, Wang and colleagues (2010) demonstrated that, instead of universal pleiotropy, ‘most genes affect a small fraction of traits whereas genes affecting more traits have larger per-trait effects’. Thus, because genetic effects on single traits—if they exist—are drastically smaller than genetic effects on multitraits, the results of quantitative genetic analyses—specifically, quantitative trait locus (QTL) analyses—would first identify loci with major pleiotropic effects.

Many pleiotropic hotspots controlling the variation of plant life history, morphology, metabolism, and physiology have been identified in natural and crop species (McKay *et al.*, 2003; Juenger *et al.*, 2005; Keurentjes *et al.*, 2006; Fu *et al.*, 2009; Haselhorst *et al.*, 2011; Edwards *et al.*, 2012; Fournier-Level *et al.*, 2013). Pleiotropic QTLs can have similar effects in different environments (no G×E), or variable effects depending of the environment (G×E), including conditional neutrality (i.e. a QTL triggers trait variation in one environment and not in another) and antagonistic pleiotropy (i.e. a QTL triggers opposite trait variation in two different environments) (El-Soda *et al.*, 2014). However for practical reasons, quantitative genetic analyses generally have focused on a limited number of environments and/or a limited number of traits. Most often they focused on traits related to resource allocation (e.g. partitioning of biomass among organs) and ignored the traits related to resource acquisition and fluxes (e.g. photosynthetic and transpiration rates) despite their importance for plant adaptation. Recently, two major pleiotropic genes, *CRY2* and *HUA2*, have been identified in *A. thaliana* to control the trade-off between the rate of carbon acquisition and plant longevity (Vasseur *et al.*, 2012); that is, a trait combination participating in the strategies of resource economics (Wright *et al.*, 2004). Nonetheless, the effect of environmental perturbations on this relationship and its genetic determinism is still unknown (Atkinson *et al.*, 2010).

The objectives of the present study were: (i) to evaluate how trait values and trait correlations vary in response to WD and HT; and (ii) to map the loci that control the integrated phenotypic responses of plants through pleiotropic G×E effects. It was found that three major loci that control flowering time have antagonistic pleiotropic effects on carbon acquisition depending on the temperature but not watering. In addition, one locus controlled the response to specific environmental combinations of the traits related to carbon acquisition independently of plant size or transpiration. Thus, the genetic control of different adaptive strategies to cope with HT and WD was identified. The findings (i) support the idea that stabilizing selection may operate on flowering time genes to minimize the cost of their antagonistic pleiotropy on WUE; and (ii) suggest that size-independent genetic effects are preferential targets to improve plant performance under WD and HT.

## Materials and methods

### Plant material and growth conditions

The Landsberg *erecta* (L*er*)×Cape Verde Islands (Cvi) population (NASC code N22000) of *A. thaliana* homozygous recombinant inbred (RI) lines (Alonso-Blanco *et al.*, 1998) was selected because previous mapping studies have shown that this population carries segregating alleles with strong pleiotropic effects (Fu *et al.*, 2009; Vasseur *et al.*, 2012) and because the parental lines exhibit contrasting responses to WD and HT (Vile *et al.*, 2012). A total of 120 RI lines (*n*=4) selected from the entire population and the parents (*n*=12) were grown in four experiments: control temperature (CT)×well-watered (WW), CT×WD, HT×WW, and HT×WD. The experiments were performed using the PHENOPSIS facility that allows automated measurements of rosette area of 504 plants under highly controlled environmental conditions (Granier *et al.*, 2006; Supplementary Fig. S1 available at *JXB* online). Plants were grown in a 12/12h day/night photoperiod in four blocks of 126 individual pots (120 RI lines plus three individuals for each of the two parental lines), all randomly distributed within each block.

Five seeds were sown at the soil surface in 225ml culture pots filled with a mixture (1:1, v:v) of loamy soil and organic compost (Neuhaus N2). Soil water content was controlled before sowing, allowing the automatic adjustment to the target soil water content by weighing and watering each pot once a day (Granier *et al.*, 2006; Supplementary Fig. S1B at *JXB* online). Between germination and the emergence of the first two true leaves, plants from all experiments were cultivated as follow: 20 °C with a daily cycle of 12h light supplied from a bank of HQi lamps which provided 190 μmol m^–2^ s^–1^ photosynthetic photon flux density (PPFD) at plant height; air water vapour pressure deficit (VPD_air_) maintained constant at 0.4–0.5 kPa, and soil moisture at 0.35g H_2_O g^–1^ dry soil. At the appearance of the cotyledons, one plant was kept per pot (additional plants were manually removed). The WD and HT treatments were applied after emergence of the first two true leaves, avoiding early growth effects. In all conditions, PPFD was maintained at 190 μmol m^–2^ s^–1^ and VPD_air_ was set to 0.7–0.8 kPa. CT was set to 20/17 °C day/night, while HT was set to 30/25 °C day/night. In natural conditions, 30 °C is one of the highest temperature encountered by *A. thaliana*, and this temperature has been identified to be the basal thermotolerance, namely the highest temperature tolerated by *A. thaliana* when plants have never encountered previous HT (Ludwig-Muller *et al.*, 2000). Soil water content was maintained with a modified one-tenth-strength Hoagland solution at 0.35g H_2_O g^–1^ dry soil under WW (corresponding to 0.07MPa soil water potential; WP4-T dewpoint meter; Decagon Devices, Pullman, WA, USA) and 0.20g H_2_O g^–1^ dry soil under WD (corresponding to 0.28MPa soil water potential; see Supplementary Fig. S2). The latter has been shown to decrease leaf water potential significantly and impair plant growth (Granier *et al.*, 2006). All environmental data, including daily soil water content, air temperature, and VPD_air_, were recorded in the course of time and are available in the PHENOPSIS database (Fabre *et al.*, 2011).

Near-isogenic lines (NILs) were also selected to confirm the QTLs identified from the genetic analysis. NILs were chosen from the population previously developed by introgressing genomic regions of Cvi into L*er* (Keurentjes *et al.*, 2007). The NIL LCN 1–2.5 (NASC code N717045; Cvi-*CRY2*_L*er*_) carries a Cvi fragment at the top of chromosome 1 and was selected to confirm the *CRY2* locus. LCN 5–6 (N717122; Cvi-*GH.473C*_L*er*_) carries a Cvi fragment in the middle of chromosome 5 and was selected to confirm the *GH.473C* locus. LCN 2–20 (N717091; Cvi-*MSAT2.22*_L*er*_) carries a Cvi fragment in the end of chromosome 2 and was selected to confirm the *MSAT2.22* locus. The NILs were grown in CT×WW in a separate experiment (for all lines, *n*=10), and measured for the same traits as the RI lines.

### Measurements of traits

#### Phenology and biomass allocation at reproduction 

Leaf growth, metabolism, and hydraulic properties change dramatically during leaf and plant development. This is notably characterized by the transition from sink to source organ, which varies among genotypes and environmental conditions (Pantin *et al.*, 2012). In this study, all traits (except growth rate) were measured at flowering (i.e. on rosettes with all vegetative leaves fully expanded) to avoid possible misleading effects of the variation in source/sink transition when developmental trajectories differ across genotypes. Age at reproduction was estimated as the number of days from sowing to opening of the first flower. At opening of the first flower, each rosette was cut, the reproductive stem was separated from the rosette, and their fresh weights were determined immediately (FW_rosette_ and FW_repro_, respectively, mg). The rosette was wrapped in moist paper and kept at 4 °C overnight in darkness. After complete rehydration, the water-saturated weight of the rosette was determined (SFW_rosette_, mg). Leaf blades were then separated from the petioles and scanned to determine the total leaf area (TLA, cm^2^). Leaf blades, petioles, and reproductive stem were then separately oven-dried at 65 °C for 96h, and their dry mass was determined. Vegetative dry mass at reproduction (DM_rosette_, mg) was calculated as the sum of dry mass of petioles (DM_petioles_, mg) and blades (DM_blades_, mg).

#### Leaf morphology and stomata density 

Traits were measured at opening of the first flower. Leaf dry mass per area (LMA, g m^–2^) was calculated as the ratio of DM_blades_ and TLA. Leaf relative water content (RWC, %) was estimated as the proportion of water in the rosette compared with the maximum weight of water when saturated: RWC=(FW_rosette_–DM_rosette_)/(SFW_rosette_–DM_rosette_). An imprint of the adaxial epidermis of the sixth leaf was obtained with a coat of varnish spread on the leaf surface. Mean adaxial stomatal density (stomata mm^–2^) was determined in two 0.12mm^2^ zones located at the bottom and at the top of the leaf from the epidermal imprints placed under a microscope (Leitz DM RB, Leica, Wetzlar, Germany) coupled to an image analyser (BioScan-Optimas 4.10, Edmond, WA, USA).

#### Rosette-level relative growth rate, transpiration, net photosynthesis, and WUE 

The rosette expansion rate was estimated using the daily zenithal images of the plants acquired within the PHENOPSIS automaton (Sony SSC-DC393P camera; Supplementary Fig. S1 at *JXB* online). The total projected leaf area of the rosette (RA, cm^2^) was determined every 2–3 d (ImageJ 1.43C, Rasband, Bethesda, MD, USA), and the relative growth rate (RGR, mg d^–1^ mg^–1^) was calculated as the derivative of the quadratic function linking the absolute growth rate *G* (mg d^–1^) to rosette dry mass (RGR=d*G*/dDM_rosette_) following Vasseur *et al.* (2012).

Measurements of whole-plant transpiration, by daily weighing of each pot, started when flower buds were macroscopically visible (bolting stage) and lasted four consecutive days. Soil evaporation was prevented by sealing the soil surface with four layers of a plastic ﬁlm (Supplementary Fig. S1A at *JXB* online). The absolute transpiration rate (*T*, mg H_2_O d^–1^) was estimated as the slope of the linear regression between pot weight and time. The transpiration rate was expressed on a rosette area basis (*T*_area_, mg H_2_O cm^–2^ d^–1^) and on a blade dry mass basis (*T*_mass_, mg H_2_O mg^–1^ d^–1^). Photosynthesis (*A*, nmol CO_2_ s^–1^) was measured measured on the vegetative rosette (after cutting the inflorescence) under growing conditions using a whole-plant chamber prototype designed for *A. thaliana* by M. Dauzat (INRA, Montpellier, France) and K.J. Parkinson (PP System, UK) and connected to an infrared gas analyser system (CIRAS 2, PP systems, USA). The photosynthetic rate was expressed on a blade dry mass basis (*A*_mass_, nmol CO_2_ g ^–1^ s^–1^) and on a blade area basis (*A*_area_, nmol CO_2_ cm^–2^ s^–1^). WUE (nmol CO_2_ mg^–1^ H_2_O) was determined as the ratio between whole-plant net photosynthesis and whole-plant transpiration (*A*/*T*).

Genotypic information, plant images, and phenotypic trait values are available in the PHENOPSIS database (http://bioweb.supagro.inra.fr/phenopsis/; Fabre *et al.*, 2011).

### Genetic analysis of multivariate plant plasticity

#### Decomposition of the variance–covariance matrix into principal components across and within environments 

Twelve morphological and physiological traits were investigated: vegetative and reproductive dry masses, age at reproduction, TLA, LMA, RWC, stomatal density, *A*_mass_, *A*_area_, *T*_mass_, *T*_area_, and RGR. Mean and standard deviation values are given in Supplementary Table S1 at *JXB* online. The coefficients of phenotypic correlation between traits were estimated as the Pearson’s product moments in each environmental condition. The coefficients of genetic correlation were estimated by dividing the covariance between RI line means for each pair of traits by the product of the square roots of among-line variance components for each trait (Supplementary Fig. S3).

A joint analysis of the geometry of the variance–covariance matrix across and within environments was performed with a dual multiple factor analysis (DMFA) including the 12 traits measured on all the individuals. DMFA is a multiple factor analysis applied to different sets (i.e. the four environments) of individuals described by the same set of variables (for details, see Abdi *et al.*, 2013). While similar to classical principal components (PCs) analysis, DMFA takes into account the internal grouping structure to decompose the eigenvectors and eigenvalues of the matrix across and within groups of individuals, and allows the superimposed representation of clouds of points from different groups in a global space. DMFA allowed representation on a unique map of the relative contribution of traits to PCs as well as the correlation between PCs across environments. Analyses were performed in the R 2.12 environment (R Development Core Team, 2009) with the DMFA function from the R/*FactoMineR* package.

#### QTL mapping of G and G×E effects on the **G**-matrix 

The additive and non-additive genetic and environmental effects on each trait and the **G**-matrix were estimated using a mixed-effects model (R/*lme4* package) fitted either on individual trait values or on the coordinates of the individuals along the first three PCs of the DMFA. Each phenotypic trait and PC was modelled as:

$${\text{P}}_{iwt}={\text{G}}_{i}+{\text{W}}_{w}+{\text{T}}_{t}+{\text{W}}_{w}\times {\text{T}}_{t}+{\text{G}}_{i}\times {\text{W}}_{w}+{\text{G}}_{i}\times {\text{T}}_{t}+{\text{G}}_{i}\times {\text{W}}_{w}\times {\text{T}}_{t}$$

where W_*w*_ and T_*t*_ are the fixed effects of watering *w* and temperature *t*, respectively [*w*=0.35g H_2_O g^–1^ dry soil or 0.20g H_2_O g^–1^ dry soil (WW and WD, respectively); *t*=20 °C or 30 °C (CT and HT, respectively)]. G is the genetic effect of genotype *i* (treated as random: *i*=1 to 120 RI lines). G_*i*_×W_*w*_, G_*i*_×T_*t*_, and G_*i*_×W_*w*_×T_*t*_ (treated as random) are the specific response of genotype *i* to the environments *w* and *t*. CT×WW was used as the intercept. The 95% confidence intervals of the fixed effects were estimated with a Markov Chain Monte Carlo algorithm following 1000 permutations. Variance components attributable to G and G×E effects were estimated from the random effects of the mixed-effects model.

The best linearized unbiased predictors (BLUPs) of the G and G×E effects that accounted for at least 5% of the total variance in the coordinates of the individuals in the three first PCs were extracted. The BLUPs were used for QTL analysis to determine the genomic regions that control the variation in the main dimensions of the phenotypic space. A total of 310 amplified fragment length polymorphism (AFLP) markers (Alonso-Blanco *et al.*, 1998) spanning all the *A. thaliana* genome were used to perform multiple-QTL composite interval mapping (R/*qtl* package). The 5% significance level threshold was calculated for QTL LOD scores following 1000 permutations (2.53<LOD_threshold_<2.78, function *mqmpermutation* from the R/*qtl* package). The percentage variability explained by each significant QTL (*P*<0.01) and significant epistatic interactions between QTLs (*P*<0.01) were quantified with two-way analysis of variance (ANOVA), using the markers for which LOD>LOD_threshold_ as putative QTLs. A 1.5 LOD interval for each QTL location was calculated with the function *lodint* from the R/*qtl* package.

#### Effect of QTLs on the phenotypic traits 

The pairwise comparison of the effects of each parental allele (L*er* or Cvi) at the QTLs *CRY2*, *MSAT2.22*, and *FD.98C* on the phenotypic traits was performed with one-way ANOVA after log_10_ transformation of the data. In addition, WUE was modelled as a quadratic function of vegetative dry mass. Since vegetative dry mass showed departure from log normality, a generalized linear model (with the *glm* function in R, using a Gaussian distribution of the error) was used as:

$$\text{WUE}=\text{a}+{\text{b}}_{wt}\times \text{DM}+{\text{c}}_{wt}\times {\text{DM}}^{\text{2}}$$

Residuals were extracted, and the effects of L*er* and Cvi alleles at *MSAT2.22* in each environment were estimated following planned pairwise comparisons after ANOVA. The confirmation of the QTL effect with the phenotypic analysis of the NILs was analysed with one-way ANOVA followed by a post-hoc Tukey test.

## Results

### Individual traits were highly variable and highly plastic to HT and WD

The 12 morphological and physiological traits were highly variable and they varied more within than between environments (see Supplementary Table S1 at *JXB* online for summary statistics and Supplementary Fig. S4 for trait distributions). Globally, HT and WD had additive effects (i.e. the specific effects of HT or WD were not dependent on the level of the other factor) on size-related traits (e.g. flowering time, TLA, or vegetative dry mass) and interactive effects on the other traits (Table 1; Supplementary Fig. S3). Among genotypes, vegetative dry mass spanned three orders of magnitude in each condition, and was on average significantly reduced by 70% (from 6% to 91% in individual genotypes) under HT and by 31% under WD (from a 77% decrease to up to a 3-fold biomass increase in five genotypes due to delayed flowering) compared with CT×WW. The combination of HT and WD reduced vegetative dry mass by 81% on average (from 19% to 96%). The negative effects of both HT and WD on plant size were also reflected in the variation of TLA and reproductive dry mass. In contrast, age at reproduction, LMA, and stomatal density were all significantly reduced under HT but increased under WD (Table 1). Inversely, RGR and transpiration rate (*T*_mass_ and *T*_area_) were increased by HT, but reduced by WD. Finally, the net photosynthetic rate (*A*_mass_ and *A*_area_) was significantly reduced by both HT and WD and more strongly by their combination. Within each environment, the genetic effects (G) explained between 34% and 88% of trait variability, except for net photosynthetic rate and RWC, for which G explained <7.5% of the variability (Table 1). Inversely, there were important variance components attributable to G×E for the net photosynthetic rate (G×T >30% and G×T×W >10%). QTL analyses revealed that only a few loci with strong pleiotropic effects explained most of the trait variation within each environment (Supplementary Fig. S5). The contribution of allelic variability to phenotypic variation was also depicted by the similarity between the phenotypic and genetic variance–covariance matrices (Supplementary Fig. S3).

**Table 1.** Coefficients and variance components of mixed-models testing the effects of the genotype (G), temperature (T), and watering regime (W) on 12 traitsEach phenotypic trait P_*iwt*_ was modelled, after log_10_ transformation, as: P_*iwt*_=W_*w*_+T_*t*_+W_*w*_×T_*t*_+G_*i*_+G_*i*_×W_*w*_+G_*i*_×T_*t*_+G_*i*_×W_*w*_×T_*t*_, where W and T are the fixed effects of watering *w* [0.35g H_2_O g^–1^ dry soil for well-watered (WW) and 0.20g H_2_O g^–1^ dry soil for water deficit (WD) conditions] and temperature *t* (20 °C for control temperature (CT) or 30 °C for high temperature (HT)), respectively; G is the genetic effect of genotype *i* (1–120 RI lines, treated as random; *n*=4); and G×W, G×T, and G×T×W are the interactive effects (treated as random).

Trait	Fixed effects				Variance components (%)			
	Intercept	WD effect	HT effect	HT×WD effect	G	G×W	G×T	G×T×W
Age at reproduction (d)	1.59 [1.58;1.61]	0.08 [0.07;0.09]	–0.1 [–0.11;–0.09]	–0.01 [–0.02;0]	78.8	2.0	8.0	0.0
Vegetative dry mass (mg)	1.44 [1.39;1.49]	–0.20 [–0.23;–0.17]	–0.63 [–0.68;–0.59]	0.01 [–0.04;0.05]	87.7	0.0	4.2	1.3
Reproductive dry mass (mg)	1.01 [0.97;1.04]	–0.27 [–0.3;–0.24]	–0.58 [–0.61;–0.56]	0.03 [0;0.07]	59.3	3.5	3.1	4.1
Total leaf area (cm^2^)	3.01 [2.97;3.06]	–0.33 [–0.36;–0.3]	–0.48 [–0.53;–0.45]	0.02 [–0.02;0.06]	86.3	0.1	4.4	2.1
Leaf mass per area (LMA, g m^–2^)	1.36 [1.35;1.38]	0.13 [0.12;0.15]	–0.18 [–0.2;–0.17]	–0.03 [–0.05;–0.01]	63.5	2.3	7.9	1.6
Relative water content (RWC, %)	1.87 [1.86;1.87]	–0.04 [–0.04;–0.04]	0.07 [0.07;0.08]	–0.03 [–0.04;–0.02]	0.0	9.0	10.6	4.0
Stomatal density (st. mm^–2^)	2.28 [2.26;2.3]	0.21 [0.2;0.22]	–0.01 [–0.03;0.01]	–0.05 [–0.07;–0.03]	34.3	0.0	19.6	7.4
*A*_mass_ (nmol CO_2_ s^–1^ g^–1^)	2.24 [2.2;2.28]	–0.27 [–0.3;–0.24]	–0.04 [–0.08;0.01]	–0.21 [–0.26;–0.16]	7.4	1.8	33.9	5.4
*A*_area_ (nmol CO_2_ s^–1^ cm^–2^)	–0.39 [–0.42;–0.36]	–0.14 [–0.17;–0.11]	–0.23 [–0.27;–0.19]	–0.23 [–0.28;–0.18]	0.0	0.0	25.1	8.7
*T*_mass_ (mg H_2_O d^–1^ mg^–1^)	1.75 [1.71;1.79]	–0.35 [–0.38;–0.32]	0.58 [0.55;0.61]	0.19 [0.15;0.23]	71.5	2.2	5.6	0.0
*T*_area_ (mg H_2_O d^–1^ cm^–2^)	2.11 [2.08;2.14]	–0.22 [–0.25;–0.19]	0.4 [0.37;0.43]	0.16 [0.13;0.2]	56.4	2.4	7.7	0.0
RGR (mg d^–1^ mg^–1^)	0.77 [0.76;0.79]	0.01 [0;0.03]	0.1 [0.1;0.12]	–0.07 [–0.09;–0.06]	37.9	36.9	12.6	5.7

*A*, net photosynthetic rate; *T*, transpiration rate.

95% confidence intervals (in brackets) were estimated with a Markov Chain Monte Carlo algorithm following 1000 permutations.

CT×WW was used as the intercept.

### The geometry of the phenotypic space differed across and within environments

**Fig. 1.**
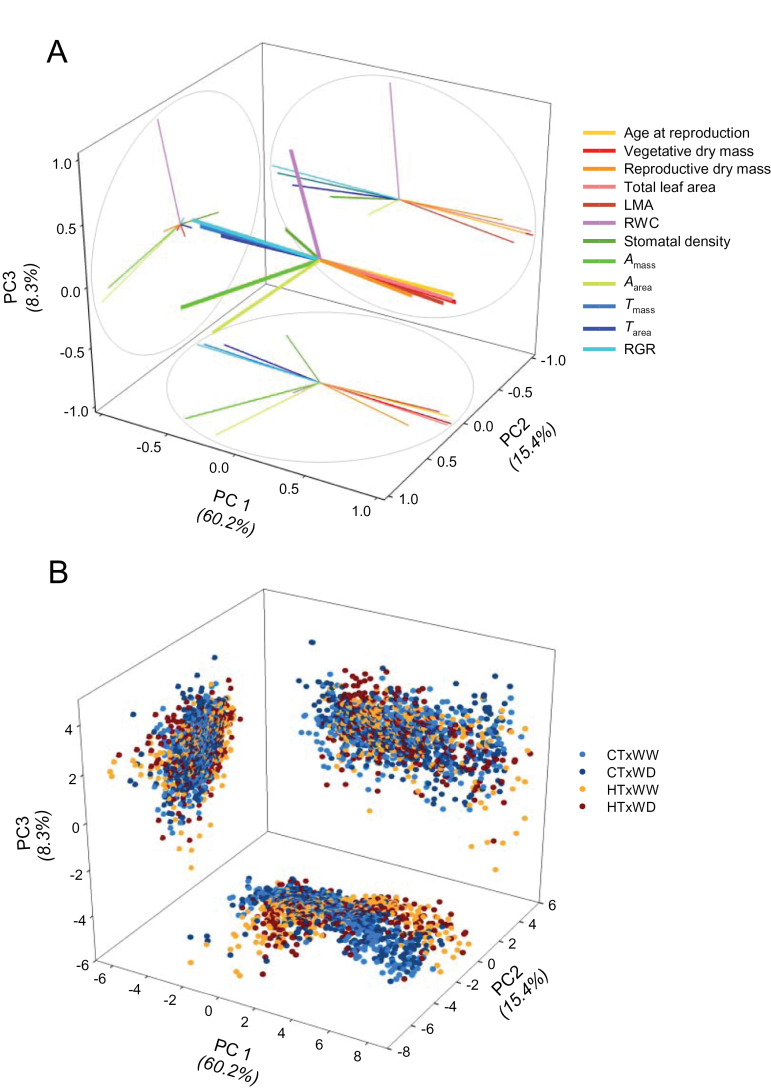
Dual multiple factor analysis (DMFA) of the phenotypic space of L*er*×Cvi recombinant inbred lines across environments. Three-dimensional representations of (A) the variables (see Table 1 for variable names) and of (B) the individuals in the first three PC spaces. CT, control air temperature (20 °C); HT, high air temperature (30 °C); WW, well-watered (0.35g H_2_O g^–1^ dry soil); WD, soil water deficit (0.20g H_2_O g^–1^ dry soil). Light blue, CT×WW; dark blue, CT×WD; orange, HT×WW; dark red, HT×WD.

Across environments, the first three PCs of the DMFA performed on the 12 traits together explained 84% of the total variance in the phenotypic space (PC1, PC2, and PC3 explained 60.2, 15.4, and 8.3%, respectively). Two sets of negatively correlated traits contributed most to PC1 (Fig. 1A; see Supplementary Tables S2 and S3 at *JXB* online for correlations of traits with PCs and trait contributions to each PC). The first set was composed of traits positively associated with PC1: plant size (vegetative and reproductive dry mass, TLA), age at reproduction, and LMA. The second set was composed of traits negatively associated with PC1: transpiration rates (*T*_mass_ and *T*_area_), RGR, and to a lesser extent stomatal density. Net photosynthetic rates (*A*_mass_ and *A*_area_) were positively correlated to PC2, as well as stomatal density, to a lesser extent. The projections of the individuals in the PC1–PC2 plane differed strongly depending on temperature (Fig. 1B). RWC contributed most to the phenotypic variability along PC3 (*r*=0.95; Supplementary Table S2).

**Fig. 2.**
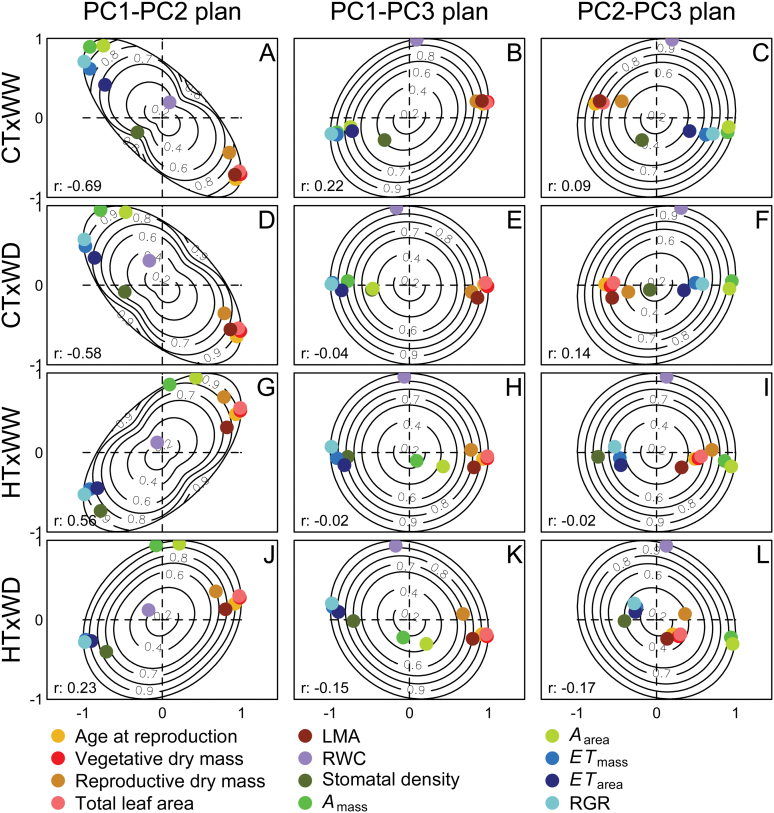
Dual multiple factor analysis (DMFA) of the phenotypic space of L*er*×Cvi recombinant inbred lines within environments. (A–L) Representation of the trait loadings on principal components PC1, PC2, and PC3. CT, control air temperature (20 °C); HT, high air temperature (30 °C); WW, well-watered (0.35g H_2_O g^–1^ dry soil); WD, soil water deficit (0.20g H_2_O g^–1^ dry soil). Contour lines represent the quality of the representation of the variables in the plan. *r* is the coefficient of correlation between PCs within each environment (note that they are orthogonal across environments).

The internal structure of trait covariations within each environment is plotted in Fig. 2 and pairwise correlations are presented in Supplementary Fig. S3 at *JXB* online. As expected, strong positive correlations between size-related traits were found in each condition. In addition, *A*_mass_ and *A*_area_ were strongly correlated to each other, as well as *T*_mass_ and *T*_area_, indicating no effect of area- or mass-based normalization on the relationships observed. Air temperature had a significant effect on the structure of trait covariations, leading to a correlation between PC1 and PC2 in opposite directions under CT (Fig. 2A, D) and HT (Fig. 2G, J). The change in the correlation between PC1 and PC2 resulted from the opposite contribution of net photosynthetic rate to PC1 between CT and HT: net photosynthetic rate was strongly correlated with plant age and size, leaf morphology, transpiration, and RGR under CT, whereas these correlations were weaker, or even opposite, under HT. The negative correlation between photosynthesis and transpiration in HT indicates an imbalance between water loss and carbon acquisition under HT, which suggests a decrease in WUE under HT. In addition, the changes in the correlation between PC1 and PC2 indicated that the trade-off between lifespan and photosynthetic capacities does not hold true under HT. In contrast to the net photosynthetic rate, there was a poor correlation between stomatal density and PC1 traits under CT, but a strong correlation under HT, leading notably to a positive correlation between transpiration and stomatal density. RWC did not contribute to the changes in correlation patterns depicted by PC1 and PC2 (i.e. it displayed only very weak correlations with other traits).

### Quantification and mapping G and G×E effects

The mixed-effects models fitted on the coordinates of the individuals along the first three PCs of the DMFA performed on the 12 traits revealed that a large part of the phenotypic variability along PC1 was attributable to G effects independently of the environment (G >87%), whereas only a small part (≤3%) was attributable to G×E (Table 2). In contrast, G had a low contribution to the variance along PC2 and PC3 (G <1%), whereas G×E had a relatively high contribution to the variance on these two axes (G×T=34% and 14% for PC2 and PC3, respectively; G×T×W=11% for PC2).

**Table 2.** Coefficients and variance components of mixed-models fitted on the individual coordinates on the first three principal components (PCs) of the dual multivariate factorial analysisEach PC was modelled as: PC_*iwt*_=W_*w*_+T_*t*_+W_*w*_×T_*t*_+G_*i*_+G_*i*_×W_*w*_+G_*i*_×T_*t*_+G_*i*_×W_*w*_×T_*t*_; G is the predictor of genotype *i* (four individual replicates per line and per treatment), treated as random, given the temperature T and watering regime W, two class factors, treated as fixed effects such as *t*=20 °C or 30 °C (CT and HT, respectively); *w*=0.35g H_2_O g^–1^ dry soil or 0.20g H_2_O g^–1^ dry soil (WW and WD, respectively).

	Fixed effects				Variance components			
	Intercept	WD effect	HT effect	HT×WD effect	G	G×W	G×T	G×T×W
PC1	–0.29 [–0.50;–0.02]	0.24 [+0.04;+0.40]	0.63 [+0.31;+0.77]	–0.41 [–0.61;–0.10]	87.4	0.0	3.1	1.4
PC2	–0.02 [–0.16;+0.16]	0.01 [–0.16;+0.17]	0.03 [–0.21;+0.24]	–0.10 [–0.33;+0.15]	0.0	1.2	33.7	11.1
PC3	–0.02 [–0.13;+0.10]	–0.02 [–0.13;+0.15]	–0.01 [–0.14;+0.15]	0.02 [–0.19;+0.20]	0.7	10.4	14.2	0.4

95% confidence intervals (in brackets) were estimated with a Markov Chain Monte Carlo algorithm following 1000 permutations.

CT×WW was used as the intercept.

**Fig. 3.**
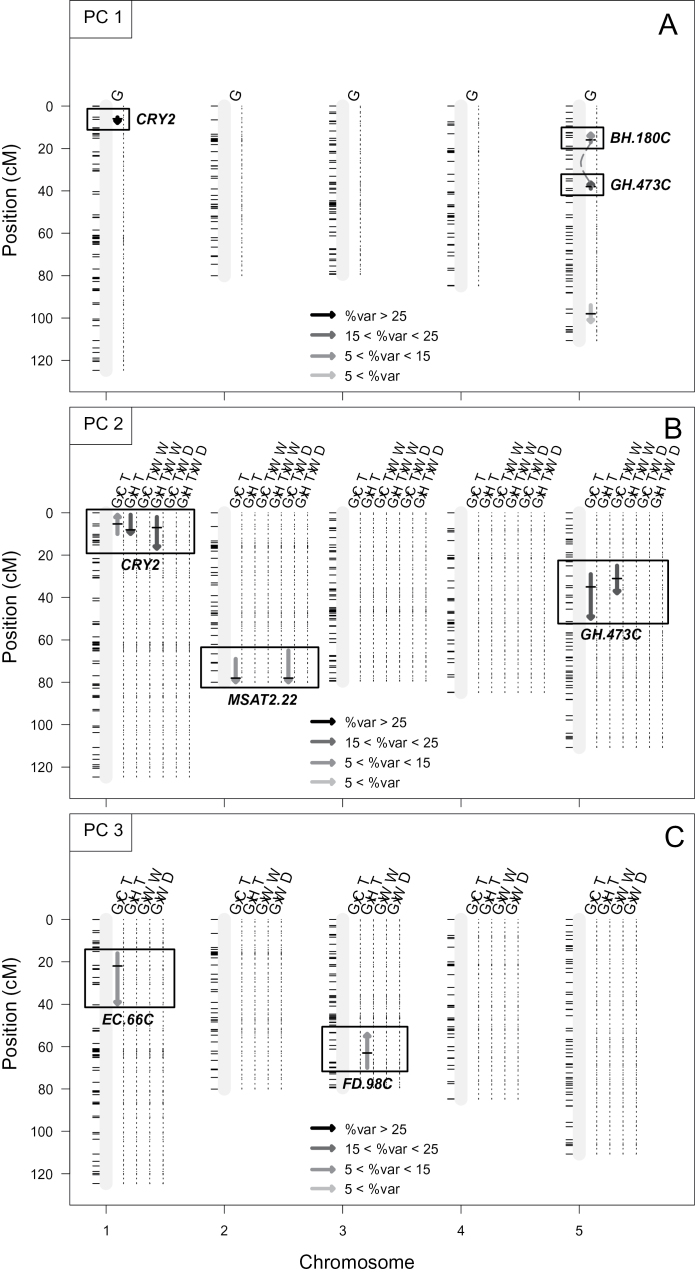
QTL mapping of genotypic (G) and genotype by environment (G×E) effects on integrated plant phenotypes. QTL mapping of the coordinates along (A) PC1, (B) PC2, and (C) PC3 from the multiple factor analysis. CT, control temperature (20 °C); HT, high temperature (30 °C); WW, well-watered (0.35g H_2_O g^–1^ dry soil); WD, water deficit (0.20g H_2_O g^–1^ dry soil). The length and the colour of the arrows represent the 1.5 LOD confidence interval of each QTL and the percentage of explained variability (0% to >25% from the clearest to the darkest), respectively. Upward and downward arrows indicate a positive or a negative effect of Cvi alleles. Only significant (*P*<0.01) QTLs and epistatic interactions between QTLs (dashed lines) are presented.

Four QTLs were associated with G effects along PC1 (all *P*<0.01; Fig. 3A; Supplementary Table S4 at *JXB* online). Among these, three had major effects: one at the top of chromosome 1 (at *CRY2* marker) explained 32% of the variance, and two epistatic QTLs closely located on chromosome 5 (at *BH.180C* and *GH.473C* markers) together explained >35% of the variance (including epistatic effects; *P*<0.001). *CRY2* and *GH.473C* also controlled the variation on PC2, but their effects depended on the environmental conditions: the Cvi alleles at *CRY2* had a positive effect on the position along PC2 under CT but a negative effect under HT (Fig. 3B; Supplementary Table S4). This result illustrated the antagonistic effect of *CRY2* on carbon assimilation (the main PC2 trait) depending on air temperature. The negative effect under HT was more pronounced when plants were well watered (HT×WW). *GH.473C* had opposite G×E effects on photosynthesis: the Cvi alleles had a negative effect on the position along PC2 under CT but no effect under HT. Thus, because of opposite and additive effects of *CRY2* and *GH.473C* on both PC1 and PC2, the L*er*/Cvi and Cvi/L*er* allelic combinations were characterized by extreme and opposite phenotypic plasticity to HT. The smallest plants (those carrying Cvi/L*er* at *CRY2*/*GH.473C*), with the highest net photosynthetic rate under CT, remained the smallest under HT but had the lowest net photosynthetic rate. In contrast, the largest plants (those carrying L*er*/Cvi at *CRY2*/*GH.473C*), with the lowest net photosynthetic rate under CT, remained the largest of the population under HT but had the highest net photosynthetic rate of the population. These temperature effects are illustrated in the shape of the phenotypic spaces (HT versus CT) projected on the PC1–PC2 plane (Fig. 1B).

A QTL at the end of chromosome 2 (*MSAT2.22*) explained >12% of the PC2 variation exclusively (no effect on PC1), but with different G×E under WD and HT. The Cvi alleles at *MSAT2.22* had a negative effect on the position along PC2 under CT and no effect under HT, indicating conditional neutrality to temperature. In addition, the effect of *MSAT2.22* observed under CT was larger under WD, illustrating G×E with both watering and temperature.

Two other QTLs also controlled PC3 variation with an interaction with temperature: *EC.66C* on chromosome 1 explained 13.3% of variability under CT, and *FD.98C* on chromosome 3 explained 12.7% of variability under HT.

All QTL effects identified in CT×WW were confirmed with the analysis of the selected NILs grown in CT×WW (Supplementary Table S5 at *JXB* online).

### QTL effects on plant development and water use efficiency

**Fig. 4.**
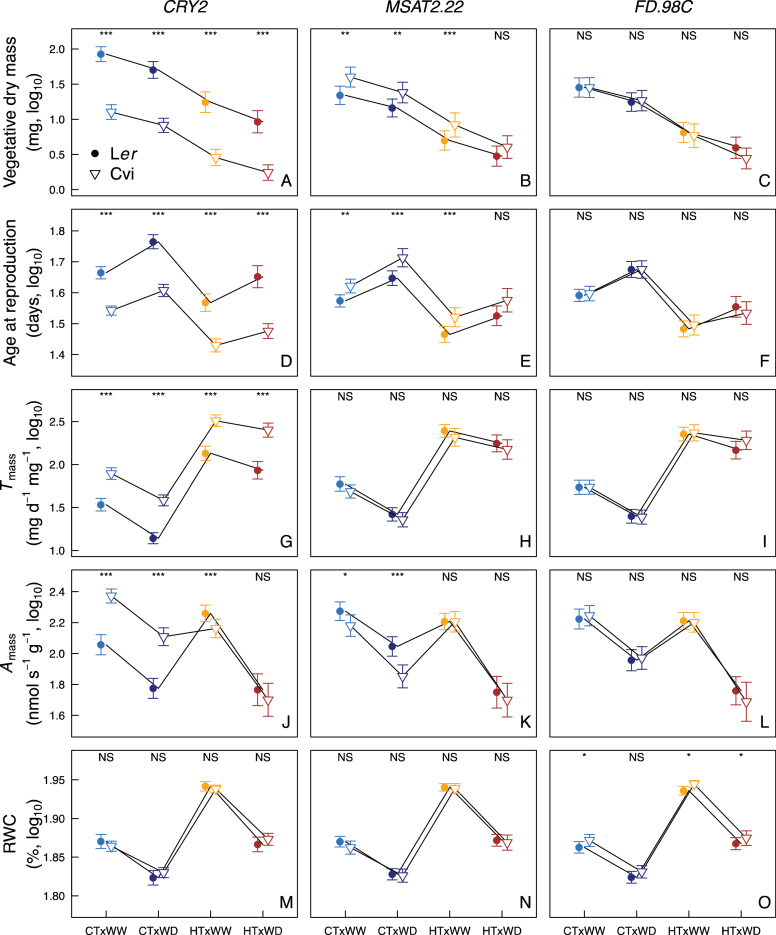
Allelic effects of selected QTLs on reaction norms of traits under contrasting temperature and watering treatments. Values of (A–C) vegetative dry mass (mg), (D–F) age at reproduction (d), (G–I) mass-based transpiration rate (*T*_mass_, mg H_2_O d^–1^ mg^–1^), (J–L) mass-based net photosynthetic rate (*A*_mass_, nmol CO_2_ s^–1^ g^–1^), and (M–O) relative water content (RWC, %) depending on the alleles (L*er*, circles; Cvi, inverted triangles) at *CRY2*, *MSAT2.22*, and *FD.98C*, respectively. CT, control temperature (20 °C); HT, high temperature (30 °C); WW, well-watered (0.35g H_2_O g^–1^ dry soil); WD, water deficit (0.20g H_2_O g^–1^ dry soil). Error bars represent 99.9% confidence intervals. Significance levels of planned pairwise comparisons for allelic effect within each treatment following two-way ANOVA: ***P<0.001; **P<0.01; *P<0.05; NS, not significant. At *CRY2*, 51 and 69 lines carry L*er* and Cvi alleles, respectively (*n*=3–4 for each line); at *MSAT2.22*, 69 and 51 lines carry L*er* and Cvi alleles, respectively (*n*=3–4 for each line); at *FD.98C*, 66 and 54 lines carry L*er* and Cvi alleles, respectively (*n*=3–4 for each line). (This figure is available in colour at *JXB* online.)

Looking at the reaction norms of individual traits in response to HT and WD (Fig. 4; Supplementary Fig. S6 at *JXB* online) allowed completion of the picture of G and G×E effects on the multivariate phenotypic space. The large effect of *CRY2* on the variation on PC1 is consistent with its effects on age at reproduction, vegetative and reproductive dry mass, LMA, and *T*_mass_ whatever the environmental conditions: *CRY2* controlled the same variation of these traits in all environments (Fig. 4A, D, G; *P*<0.001). Conversely, the effects of *CRY2* on *A*_mass_, a PC2 trait, depended on the environment, mainly on the temperature: *CRY2* had strong effects on the net photosynthetic rate under CT but not under HT (Fig. 4J), while it had low effects on stomatal density under CT but strong effects under HT (Supplementary Fig. S6). Consistent with the analysis of the coordinates along PC2, we found contrasting effects of *MSAT2.22* on *A*_mass_ in response to both soil water content and air temperature: *MSAT2.22* had a strong effect under CT and even stronger in CT×WD (Fig. 4K). Consistent with the analysis of PC3, no effect of *CRY2* and *MSAT2.22* on RWC was found whatever the environment (Fig. 4M, N). Finally, *FD.98C* had no effect on age and size at reproduction, transpiration, net photosynthetic rate (Fig. 4C, F, I, L), and stomatal density (Supplementary Fig. S6), but this QTL had significant effects on RWC in all environments except CT×WD (Fig. 4O).

**Fig. 5.**
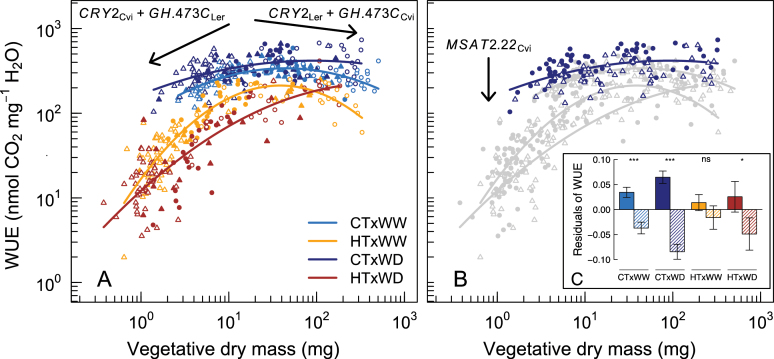
Allelic effects of *CRY2*, *GH.473C*, and *MSAT2.22* on the relationship between WUE and vegetative dry mass. WUE was modelled as a quadratic function of vegetative dry mass with generalized linear model (*glm* function in R) as: WUE=a+b_*wt*_×DM+c_*wt*_×DM^2^. (A) Projection of the individuals according to their allelic combination at *CRY2* and *GH.473C* (filled triangles, Cvi/Cvi; filled circles, L*er*/L*er*; open triangles, Cvi/L*er*; open circles, L*er*/Cvi at *CRY2*/*GH.473C*, respectively). Arrows depict the additive effects of the L*er* and Cvi alleles at *CRY2* (*CRY2*_L*er*_ and *CRY2*_Cvi_, respectively) and *GH.473C* (*GH.473C*_L*er*_ and *GH.473C*_Cvi_, respectively). Curves are the quadratic fits in each environment. CT, control temperature (20 °C); HT, high temperature (30 °C); WW, well-watered (0.35g H_2_O g^–1^ dry soil); WD, water deficit (0.20g H_2_O g^–1^ dry soil). (B) Projection of the individuals according to the allele at *MSAT2.22* in CT×WD (filled circles, L*er* alleles; open triangles, Cvi alleles). The arrow depicts the effect of Cvi alleles at *MSAT2.22* (*MSAT2.22*_L*er*_). (C) Effects of L*er* (solid bars) and Cvi (dashed bars) alleles at *MSAT2.22* on the residuals of the quadratic function in each environment [69 and 51 lines carrying L*er* and Cvi alleles, respectively (*n*=3–4 for each line)]. Error bars represent standard errors. Significance levels of planned pairwise comparisons for allelic effect within each treatment following two-way ANOVA: ***P<0.001; **P<0.01; *P<0.05; NS, not significant. (This figure is available in colour at *JXB* online.)

Because of opposite and additive effects, and independent of the environment, *CRY2* and *GH.473C* generated a negative relationship between plant size and transpiration rate in all environments. In addition, *CRY2* and *GH.473C* had opposite and additive G×E effects (mainly with temperature) on the net photosynthetic rate. It was therefore investigated how these QTLs controlled WUE in response to HT. Modelling the relationship between WUE and vegetative dry mass revealed a convex relationship that indicated optimum WUE for intermediate plant size (Fig. 5A). The non-linearity was enhanced under stressful conditions, especially under HT (Fig. 5A; Supplementary Table S6 at *JXB* online). This result illustrated a strong decrease of WUE for the small plants under HT, and a small decrease of WUE for the large plants under HT (both were non-parental allelic combinations). On the other hand, the effect of *MSAT2.22* on WUE was investigated since this QTL impacted only the net photosynthetic rate, but differently across environments. As expected, *MSAT2.22* controlled size-independent variations of WUE. However, the *MSAT2.22* effects on WUE were specific to some combinations of air temperature and watering: the effects were strongly decreased under HT and slightly increased under WD (Fig. 5B, C). Consequently, there was no significant effect of *MSAT2.22* on WUE under HT×WW.

## Discussion

### Non-adaptive responses of plant development, leaf morphology, growth dynamics, and transpiration

Our multivariate modelling approach revealed that HT, WD, and HT×WD had fixed effects on the first PC axis that combined plant size, age at reproduction, LMA, RGR, and transpiration rate. The fixed effects of HT, WD, and HT×WD represent the average environmental effects on trait values across the population, which are thought to be non-adaptive responses because they are common to all genotypes. Fixed effects on PC1 traits suggested that the plasticity of plant development and phase change is a major response strategy, shared and conserved between genotypes to minimize the impact of HT and WD on physiology, growth, and reproduction. Inspection of individual traits showed that the HT×WD effect on PC1 was mainly due to non-additive responses of transpiration rate and RGR to the combination of HT and WD. This indicates that growth and transpiration had responses to the combined stresses that were not the sum of the individual stress effects. This can be explained by the competing demand of water evaporation for cooling and water saving for growth. In contrast, age and size at reproduction and LMA exhibited purely additive fixed HT and WD effects, in accordance with what has been recently observed in a set of natural accessions of *Arabidopsis* (Vile *et al.*, 2012). This result suggests that plant development and biomass allocation are similarly constrained by water availability whatever the temperature, and vice versa.

Both HT and WD reduced plant size, although there were important differences in the response of the other traits. Development and flowering time were delayed under WD, similarly to what has been observed in *A. thaliana* (Tisné *et al.*, 2010; Vile *et al.*, 2012; Bresson *et al.*, 2013) and commonly found in natural and crop species (Barnabas *et al.*, 2008; McMaster *et al.*, 2009). Delayed reproduction, and more generally a reduced developmental rate, can result from the reduction of metabolic rates due to moderate resource limitation. Conversely, shortening of developmental phases in response to increasing temperature was observed, as expected due to temperature-mediated activation of metabolic processes (Parent *et al.*, 2010), and commonly observed in different species (Balasubramanian *et al.*, 2006; Barnabas *et al.*, 2008). Accordingly, RGR was increased and plants flowered earlier but were smaller under HT.

As expected for a water-saving strategy, the transpiration rate was on average decreased under WD. On the other hand, the transpiration rate was increased under HT, which could reflect a physiological adaptation to avoid overheating of the photosynthetic tissues (Crawford *et al.*, 2012), or a side effect of the altered carbon status on leaf geometry such as increased petiole length and leaf inclination (Vasseur *et al.*, 2011), which were both observed under HT (Supplementary Fig. S7 at *JXB* online). It is noteworthy that the plasticity in transpiration rate was coordinated with the plasticity in LMA and RGR, but not in stomatal density. This indicates that transpiration is intimately linked to growth and leaf structure, but that stomatal size and opening are probably more important than density to regulate the transpiration rate.

### Adaptive response to temperature caused by the antagonistic pleiotropy of flowering time QTLs on WUE

The QTLs at *CRY2*, *GH.473C*, and *BH.180C* controlled a large part of the variation in flowering time and plant size independently of the environment (i.e. G effect on PC1). A mutant analysis in control conditions (CT×WW) revealed that the *CRY2* gene, which encodes a cryptochrome involved in light perception and flowering time, controls a fraction of the phenotypic variation observed here (Vasseur *et al.*, 2012; Supplementary Table S7 at *JXB* online). It is likely that this gene is a, if not the, major contributor of the QTLs found at the top of chromosome 1. Similarly, *HUA2*, a flowering time gene, has been shown to contribute to the *GH.473C* effect (Doyle *et al.*, 2005; Vasseur *et al.*, 2012), and *FLC* is an epistatic mutation at *BH.180C* that acts as a positive regulator of *HUA2*. However, the confidence regions for the QTLs encompassed many genes, and the present study cannot distinguish between strict pleiotropy and linkage disequilibrium as the cause of QTL co-location. If these hotspots consist of clusters of genes of major effects, this is as interesting and informative about the evolutionary process as the question of the role of strict pleiotropy.

The developmental variability driven by flowering time QTLs generated large variation of important physiological traits, including leaf structure, RGR, and transpiration rate. However, the lack of G×E in the pleiotropic effects of *CRY2*, *GH.473C*, and *BH.180C* on PC1 traits resulted in a lack of plasticity in the correlations between PC1 traits. Within each environment, large and long-lived plants with high LMA (typically those carrying the L*er*/Cvi allelic combination at *CRY2*/*GH.473C* loci) exhibited a lower transpiration rate compared with small and short-lived plants with low LMA and high RGR (typically Cvi/L*er* at *CRY2*/*GH.473C*). The lack of G×E highlights the robustness of fundamental trade-offs to major environmental stresses: the increase in flowering time and plant size is constrained at the genetic level by the decrease in growth rate and transpiration, and the increase in leaf density, or thickness. The lack of G×E also suggests that developmental plasticity is not adaptive in itself, because there are not variable developmental responses across genotypes that could be selected. Although there was a large range of developmental strategies driven by flowering time QTLs, neither *CRY2*, *GH.473C*, nor *BH.180C* had an effect on the response of development (and associated traits) to the environment. Nonetheless, these flowering time QTLs also exhibited strong G×E effects on PC2 traits. This illustrates that the response of net photosynthesis differed strongly across genotypes and, therefore, across developmental strategies. Thus, it was hypothesized that the different developmental strategies driven by the flowering time QTLs could be adaptive because of associated G×E effects on plant carbon physiology.

Carbon acquisition through photosynthesis is tightly linked to the structure and lifespan of photosynthetic organs (Kikuzawa, 1995). The so-called ‘Leaf Economics Spectrum’ (Wright *et al.*, 2004), illustrated by the trade-offs between LMA, leaf lifespan, and photosynthetic rate across species, translates the necessity to increase carbon allocation to leaf structure (LMA) to support higher leaf area and resist mechanical damage when leaf lifespan increases. In turn, higher LMA penalizes carbon acquisition because of the reduction in light interception and CO_2_ permeability (Shipley *et al.*, 2006; Flexas *et al.*, 2012). It was recently demonstrated that *CRY2* and *HUA2* control variations in carbon economy across the L*er*×Cvi population grown in control conditions (Vasseur *et al.*, 2012). On the other hand, photosynthesis is also linked to transpiration, and the resulting trade-off is generally represented through WUE. Here, it is shown that *CRY2* and *GH.473C* exhibit antagonistic pleiotropy depending on air temperature, which resulted in a strong plasticity of carbon economics and WUE. Net photosynthetic rate and PC1 traits were negatively correlated under CT (under both WW and WD), which is consistent with the relationship observed at the interspecific level between lifespan and the efficiency of the photosynthetic organ. In contrast, the net photosynthetic rate and PC1 traits were positively related under HT (under both WW and WD), which can be explained by changes in leaf orientation [i.e. hyponastic movements that allow the capture of more light (Vasseur *et al.*, 2011)]. Because L*er* and Cvi alleles at *CRY2* and *GH.473C* have additive and opposite effects, they are responsible for a strong decrease in the WUE for the smallest and largest plants in the population (both are non-parental allelic combinations; Fig. 5A). This result supports the hypothesis that the variation of plant development can be adaptive through associated G×E on plant physiology. It also suggests that improvement of plant tolerance to HT can be mainly reached by variation of plant development. Previous findings suggest that the allelic combinations at *CRY2* and *HUA2* (the most probable causal polymorphism under *GH.473C*) might have been fixed to generate intermediate plant size that optimizes the physiological trade-offs associated with growth strategies (Vasseur *et al.*, 2012). Here it is shown that the allelic combinations at *CRY2* and *GH.473C* that have been fixed in the Cvi and L*er* strains exhibited lower plasticity of WUE to HT and WD than the non-parental allelic combinations (and, thus, WUE remains higher across environments). The findings suggest that there is stabilizing selection on flowering time and plant size to avoid the deleterious effect of antagonistic pleiotropy on plant development and physiology under stressful conditions.

### Adaptive response caused by size-independent plasticity of WUE to both HT and WD

Some QTLs controlled the plasticity of traits independently of the variations in plant development and associated traits (i.e. PC1 traits). Most notably, net photosynthesis and WUE exhibited strong size-independent G×E. The plasticity of WUE is assumed to be limited because CO_2_ and water fluxes share common regulating processes, such as stomatal opening and conductance (Pantin *et al.*, 2012). Here, WUE was estimated at the whole-plant level through the ratio between the rate of carbon fixation at flowering and the transpiration rate averaged over 4 days and nights (at floral bud emergence). Changes in WUE are therefore reflecting the cumulative effects of different processes, including stomata-related traits (e.g. density, size, and conductance), and others (e.g. cuticle thickness and plant architecture). The phenotypic variation controlled by non-flowering time QTLs was limited since PC2 and PC3 represented only 15% and 8% of the total phenotypic variation, but it may have crucial consequences on plant performance. For instance, L*er* alleles at *MSAT2.22* resulted in an increased WUE in response to both CT and WD independently of plant size (Fig. 5B, C). Previous studies also identified *MSAT2.22* as involved in the plasticity of WUE estimated from carbon isotopic discrimination (McKay *et al.*, 2003; Hausmann *et al.*, 2005). Recently, Des Marais *et al.* (2014) identified a mitogen-activated protein (MAP) kinase, MPK12, as the causal polymorphism controlling variation of WUE in this population. Their results suggest that the Cvi allele at *MPK12* reduced WUE through variation of stomatal size, opening, and conductance in response to abscisic acid (ABA), a water stress signalling molecule. However, MPK12 did not drive variation of photosynthesis, suggesting that changes in WUE were more likely to be due to changes in transpiration rate. Here it is shown that *MSAT2.22* impacted mostly PC2 traits, namely stomatal density and net photosynthetic rate. Moreover, a reduction of the effect of *MSAT2.22* under HT and WW conditions compared with CT and WD conditions was shown (Fig. 5C), although HT and WD often occur simultaneously in the field. An exciting question is what are the mechanisms by which MPK12 drives the plasticity of WUE, and how these mechanisms vary in different environmental situations.

### Conclusion

The QTLs identified here as being involved in multivariate plasticity are, by definition, pleiotropic. At the heart of the theory stands the idea that major ‘genetic hubs’ would induce systemic responses to abiotic stresses (Chapin, 1991). As a consequence of pleiotropy, in many breeding programmes the selection for higher WUE has led to the selection on flowering time genes (Blum, 2009). Here it was shown that selection for high WUE under CT could retain alleles with very low WUE under HT, because of the antagonistic pleiotropy of flowering time QTLs on WUE in response to temperature. Furthermore, it was shown that improving WUE to WD could be reached without selecting for flowering time QTLs. The identified *MSAT2.22* QTL is a promising target to optimize WUE under stressful conditions. However, the size-independent WUE effects are strongly dependent on the interaction between HT and WD. Hence, the results highlight different strategies to adapt to HT and WD. In crop breeding, the fine-tuning of these different adaptive strategies would allow the definition of ideotypes targeted to specific environmental conditions.

## Supplementary data

Supplementary data are available at JXB online.

Figure S1. The PHENOPSIS automated phenotyping platform.

Figure S2. Relationship between soil water content and soil water potential.

Figure S3. Heatmap of genetic (above-diagonal) and phenotypic (below-diagonal) correlations between traits in L*er*×Cvi RI lines under WD and HT.

Figure S4. Distribution of the 12 phenotypic traits in each environment.

Figure S5. QTL analysis of nine phenotypic traits within the four environments.

Figure S6. Allelic effects of three QTLs on the reaction norms under contrasting temperature and watering treatments.

Figure S7. Examples of leaf hyponastic movements observed in response to HT.

Table S1. Summary statistics of the 12 traits in each environmental condition.

Table S2. Correlations between the phenotypic traits and the PC of the DMFA: comparison across versus within environment.

Table S3. Contribution of the phenotypic traits to each PC of the DMFA.

Table S4. QTLs for G and G×E effects on the plant phenotypic space dimensions.

Table S5. Effect of the Cvi introgressions at *CRY2*, *GH.473C*, and *MSAT2.22* in L*er* (NILs) on vegetative dry mass and WUE.

Table S6. Water use efficiency (WUE, nmol CO_2_ mg^–1^ H_2_O) modelled as a quadratic function of vegetative dry mass.

Table S7. Effect of mutations at *CRY2* and *HUA2* genes on vegetative dry mass and WUE.

Supplementary Data
