# Supplementary material for: Multivariate genetic analysis of plant responses to water deficit and high temperature revealed contrasting adaptive strategies
Source: J Exp Bot. 2014 Sep 22;65(22):6457–69. doi: 10.1093/jxb/eru364 (PMC4246181; doi:10.1093/jxb/eru364)
Supplement: Supplementary Data [file supp_65_22_6457__index.html]

Multivariate genetic analysis of plant responses to water deficit and high temperature revealed contrasting adaptive strategies — Multivariate genetic analysis of plant responses to water deficit and high temperature revealed contrasting adaptive strategies — Supplementary Data 

# Multivariate genetic analysis of plant responses to water deficit and high temperature revealed contrasting adaptive strategies

## Supplementary Data

Data files

**Files in this Data Supplement:**

- Supplementary Data - Supplementary Data
