# Supplementary material for: Multivariate genetic analysis of plant responses to water deficit and high temperature revealed contrasting adaptive strategies
Source: J Exp Bot. 2014 Sep 22;65(22):6457–69. doi: 10.1093/jxb/eru364 (PMC4246181; doi:10.1093/jxb/eru364)

Multivariate genetic analysis of plant responses to water deficit and high temperature revealed contrasted adaptive strategies

Francois Vasseur, Thibaut Bontpart, Myriam Dauzat, Christine Granier, and Denis Vile

Supplementary Data

Table S1. Summary statistics of the 12 traits in each environmental condition. Mean and standard deviation (SD) under control (CT, 20 °C) and high (HT, 30 °C) air temperatures and under well-watered (WW, 0.35 g H_20 g^{-1} dry soil) and water deficit (0.20 g H_20 g^{-1} dry soil) conditions. List of abbreviations: leaf dry mass per area (LMA, g m^{-2}), relative water content (RWC, %), mass-based net photosynthetic and transpiration rates (A_{mass}, nmol CO_2 s^{-1} g^{-1} and T_{mass}, mg H_2O d^{-1} mg^{-1}, respectively), area-based net photosynthesis and transpiration rates (A_{area}, nmol CO_2 s^{-1} cm^{-2} and T_{area}, mg H_2O d^{-1} cm^{-2}, respectively), and relative growth rate (RGR, mg d^{-1} mg^{-1}). 122 genotypes (*n* = 4 for the RI lines and *n* = 12 for the parental lines) for all traits in each condition.

	**CTxWW**		**CTxWD**		**HTxWW**		**HTxWD**	
	**mean**	**SD**	**mean**	**SD**	**mean**	**SD**	**mean**	**SD**
**Age at reproduction (d)**	40.55	9.59	49.82	14.73	32.80	11.34	37.97	15.51
**Vegetative dry mass (mg)**	76.60	105.71	52.05	80.51	28.39	57.92	14.69	34.71
**Reproductive dry mass (mg)**	13.12	8.34	6.54	3.57	3.99	3.64	1.99	1.64
**Total leaf area (cm^2)**	19.33	20.35	10.33	14.14	9.80	15.86	4.11	8.40
**Leaf mass per area (LMA, g m^{-2})**	25.16	9.68	34.43	12.52	15.75	4.88	20.15	7.63
**RWC (relative water content %)**	73.92	5.72	67.46	5.47	87.11	4.69	74.46	5.98
**Stomata density (mm^{-2})**	195.07	55.98	316.05	89.39	204.82	105.31	287.35	115.75
**A_{mass} (nmol CO_2 s^{-1} g^{-1})**	200.09	105.20	114.55	76.31	186.77	86.27	36.79	132.88
**A_{area} (nmol CO_2 s^{-1} cm^{-2})**	0.42	0.13	0.33	0.14	0.28	0.12	0.08	0.23
**T_{mass} (mg H_2O d^{-1} mg^{-1})**	72.64	64.38	34.15	32.78	309.16	256.19	242.33	202.27
**T_{area} (mg H_2O d^{-1} cm^{-2})**	145.93	89.65	89.03	49.58	415.34	288.48	392.67	281.83
**RGR (mg d^{-1} mg^{-1})**	0.76	0.17	0.79	0.01	0.89	0.23	0.83	0.11

Table S2. Correlations between the phenotypic traits and the PC of the DMFA: comparison across- versus within-environment. Age at reproduction (d), vegetative and reproductive dry masses (mg), total leaf area (cm^2), leaf dry mass per area (LMA, g m^{-2}), relative water content (RWC, %), stomatal density (mm^{-2}), mass-based net photosynthetic and transpiration rates (A_{mass}, nmol CO_2 s^{-1} g^{-1} and T_{mass}, mg H_2O d^{-1} mg^{-1}, respectively), area-based net photosynthesis and transpiration rates (A_{area}, nmol CO_2 s^{-1} cm^{-2} and T_{area}, mg H_2O d^{-1} cm^{-2}, respectively), and relative growth rate (RGR, mg d^{-1} mg^{-1}).

		PC1	PC2	PC3
**all environments**	Age at reproduction	0.9305	-0.0314	-0.0298
	Vegetative dry mass	0.9837	0.0441	-0.0333
	Total leaf area	0.9702	0.0699	-0.0029
	Reproductive dry mass	0.7605	0.1992	0.033
	Stomatal density	-0.532	-0.4615	-0.118
	RWC	-0.0867	0.2061	0.9495
	LMA	0.8521	-0.0519	-0.1322
	A_{mass}	-0.5097	0.8313	-0.1174
	A_{area}	-0.2335	0.9122	-0.195
	T_{mass}	-0.9438	-0.0705	-0.0206
	T_{area}	-0.8256	-0.1331	-0.095
	RGR	-0.9837	-0.0441	0.0333
**CTxWW**	Age at reproduction	0.9314	-0.6953	0.2322
	Vegetative dry mass	0.9856	-0.6098	0.2493
	Total leaf area	0.9718	-0.5823	0.2411
	Reproductive dry mass	0.8395	-0.3492	0.2298
	Stomatal density	-0.3029	-0.2767	-0.3268
	RWC	0.0898	0.2126	0.9611
	LMA	0.9112	-0.5989	0.2454
	A_{mass}	-0.9214	0.8282	-0.2479
	A_{area}	-0.7588	0.903	-0.2039
	T_{mass}	-0.9201	0.5049	-0.2818
	T_{area}	-0.7407	0.3315	-0.2524
	RGR	-0.9856	0.6098	-0.2493
**CTxWD**	Age at reproduction	0.9407	-0.3968	0.018
	Vegetative dry mass	0.9868	-0.295	3.00E-04
	Total leaf area	0.9662	-0.2691	0.0584
	Reproductive dry mass	0.7826	-0.1143	-0.0783
	Stomatal density	-0.4626	-0.315	-0.1189
	RWC	-0.1768	0.297	0.9463
	LMA	0.8475	-0.3016	-0.1891
	A_{mass}	-0.7892	0.8082	0.0185
	A_{area}	-0.4849	0.9037	-0.1103
	T_{mass}	-0.9668	0.1958	0.0088
	T_{area}	-0.8566	0.0828	-0.119
	RGR	-0.9868	0.295	-3.00E-04
**HTxWW**	Age at reproduction	0.9306	0.4604	-0.172
	Vegetative dry mass	0.9841	0.5025	-0.1663
	Total leaf area	0.9783	0.534	-0.1384
	Reproductive dry mass	0.7391	0.6676	-0.0515
	Stomatal density	-0.7371	-0.6996	0.0127
	RWC	-0.0611	0.1797	0.952
	LMA	0.8401	0.3058	-0.2753
	A_{mass}	-0.0074	0.87	-0.1146
	A_{area}	0.3324	0.9529	-0.2204
	T_{mass}	-0.9213	-0.4514	0.0162
	T_{area}	-0.8184	-0.4408	-0.0834
	RGR	-0.9841	-0.5025	0.1663
**HTxWD**	Age at reproduction	0.9156	0.337	-0.2241
	Vegetative dry mass	0.979	0.419	-0.2409
	Total leaf area	0.9685	0.4373	-0.2047
	Reproductive dry mass	0.6552	0.4499	0.075
	Stomatal density	-0.6874	-0.5145	-0.0383
	RWC	-0.1835	0.1334	0.9395
	LMA	0.8044	0.2482	-0.3021
	A_{mass}	-0.1371	0.8587	-0.173
	A_{area}	0.1573	0.9282	-0.2783
	T_{mass}	-0.9644	-0.3915	0.1847
	T_{area}	-0.889	-0.3971	0.1019
	RGR	-0.979	-0.419	0.2409

Table S3. Contribution of the phenotypic traits to each PC of the DMFA. The contribution of a data point to the inertia of an axis is the quotient between the inertia of its projection and the inertia of the whole scatterplot's projection on this axis. Age at reproduction (d), vegetative and reproductive dry masses (mg), total leaf area (cm^2), leaf dry mass per area (LMA, g m^{-2}), relative water content (RWC, %), stomatal density (mm^{-2}), mass-based net photosynthetic and transpiration rates (A_{mass}, nmol CO_2 s^{-1} g^{-1} and T_{mass}, mg H_2O d^{-1} mg^{-1}, respectively), area-based net photosynthesis and transpiration rates (A_{area}, nmol CO_2 s^{-1} cm^{-2} and T_{area}, mg H_2O d^{-1} cm^{-2}, respectively), and relative growth rate (RGR, mg d^{-1} mg^{-1}).

	**PC1**	**PC2**	**PC3**
Age at reproduction	11.9855	0.0532	0.0891
Vegetative dry mass	13.395	0.105	0.1113
Total leaf area	13.0294	0.2636	9.00E-04
Reproductive dry mass	8.0064	2.141	0.109
Stomatal density	3.9185	11.4915	1.3949
RWC	0.1041	2.2912	90.2982
LMA	10.05	0.1452	1.7503
A_{mass}	3.5963	37.288	1.3809
A_{area}	0.7548	44.8929	3.8068
T_{mass}	12.3304	0.2682	0.0427
T_{area}	9.4347	0.9552	0.9045
RGR	13.395	0.105	0.1113

Table S4. QTL for G and GxE effects on the plant phenotypic space dimensions. QTL mapping was performed on the BLUPs of genetic effects (G) and genotypic-by-environment (GxE) effects (*i.e.* GxT, GxW, and GxTxW for the genotypic interactions with air temperature, water availability, and their interactions, respectively). The best linearized unbiased predictors (BLUPs) estimated from mixed-effects models (that explain > 5% of variance). Brackets represent 1.5-LOD interval for each QTL location. Percent of variability was estimated with two-ways ANOVA in multiple-QTL composite interval mapping. All presented QTL are significant (*P* < 0.01). 'Marker' is the name of the closest marker to the LOD score peak.

PC	BLUPs	Marker	Chr	Position (cM)	% var
**1**	G	*CRY2*	1	6 [5.3-8]	31.9
		BH.180C	5	16 [13-17]	12.8
		GH.473C	5	38 [36-39]	20.4
		BF.168L	5	98 [94-102]	3.6
**2**	GxCT	*CRY2*	1	5.3 [1-10]	11.7
		MSAT2.22	2	78 [69-80]	12.4
		GH.473C	5	35 [29-50]	16.7
	GxHT	*CRY2*	1	8 [1-10]	16.7
	GxCTxWW	*GH.473C*	5	31 [25-38]	21.5
	GxHTxWW	*CRY2*	1	7 [2-17]	24.4
	GxCTxWD	*MSAT2.22*	2	78 [65-80]	13.2
**3**	GxCT	*EC.66C*	1	22 [16-40]	13.3
	GxHT	*FD.98C*	3	63 [54-70]	12.7

Table S5. Effect of the Cvi introgressions at *CRY2, GH.473C* and *MSAT2.22* in L*er* (NILs) on vegetative dry mass and WUE. Values are the ratio of mean phenotypic trait values from NILs grown in CTxWW in a separate experiment. Vegetative dry mass (mg) and water use efficiency (WUE, nmol CO_2 mg^{-1} H_2O). Each NIL introgressed at *CRY2* (Cvi-*CRY2*$_{Ler}$), *GH.473C* (Cvi-*GH.473C*$_{Ler}$), and *MSAT2.22* (Cvi-*MSAT2.22*$_{Ler}$) was compared to the parental lines (L*er* and Cvi). Genotypes were compared with a post-hoc Tukey test following one-way ANOVA (7 < *n* < 10). Significance codes: *** *P* < 0.001; ** *P* < 0.01; * *P* < 0.05; . *P* < 0.1.

	Compared to	Vegetative dry mass		WUE	
**L*er***	Cvi	0.88	**	1.11	***
**Cvi-CRY2$_{Ler}$**	L*er*	0.49	***	0.93	***
**Cvi-CRY2$_{Ler}$**	Cvi	0.43	***	1.03	NS
**Cvi-GH.473C$_{Ler}$**	L*er*	1.40	***	0.76	***
**Cvi-GH.473C$_{Ler}$**	Cvi	1.24	***	0.84	***
**Cvi-MSAT2.22$_{Ler}$**	L*er*	0.99	NS	0.96	**
**Cvi-MSAT2.22$_{Ler}$**	Cvi	0.88	**	1.06	***

Table S6. WUE modelled as a quadratic function of vegetative dry mass. WUE was modelled as a quadratic function of vegetative dry mass with generalized linear model (*glm* function in R) as: WUE = a + b_{wt} × DM + c_{wt} × DM^2. c, b and c are the coefficients of zero, first and second order, respectively, of the quadratic function between water use efficiency (WUE, nmol CO_2 mg^{-1} H_2O). 95 % confidence intervals (CI, in brackets) were estimated with the *confint* function in R. Significance codes: *** = $P < 0.001$; ** = $P < 0.01$; * = $P < 0.05$; ° = $P < 0.1$ (n = 450-500 in each condition). CTxWW used as intercept.

Trait	Effect	a	95% CI		b	95% CI		c	95% CI	
WUE	intercept	1.949	[1.778;2.12]	***	0.708	[0.468;0.947]	***	-0.215	[-0.291;-0.139]	***
	HT effect	-0.723	[-0.902;-0.545]	***	0.683	[0.414;0.953]	***	-0.224	[-0.317;-0.13]	***
	WD effect	0.271	[0.064;0.479]	*	-0.298	[-0.601;0.006]	.	0.11	[0.01;0.21]	*
	HTxWD effect	-0.413	[-0.633;-0.194]	***	-0.158	[-0.508;0.192]	NS	0.157	[0.026;0.289]	*

Table S7. Effect of mutations at *CRY2* and *HUA2* genes on vegetative dry mass (mg) and age at reproduction (day). Each mutant at *CRY2* ($cry2_{\mathrm{Col}}$ in Col-4 background and $cry2_{\mathrm{Ler}}$ in L*er*-0 background), and at *HUA2* ($hua2_{\mathrm{Col}}$ in Col-0 background), was compared to its respective wild-type (background). Genotypes were compared with a post-hoc Tukey test following one-way ANOVA (7 < n < 10). Significance codes: *** $P < 0.001$; ** $P < 0.01$; * $P < 0.05$; . $P < 0.1$. Data from Vasseur *et al.* (2012).

	**Compared to**	**Age at reproduction**		**Vegetative dry mass**	
**L*er***	Cvi	0.93	NS	0.69	*
**Col-0**	Col-4	0.95	NS	1	NS
$\boldsymbol{cry2_{\mathrm{Col}}}$	Col-4	1.14	***	1.36	***
$\boldsymbol{cry2_{\mathrm{L}er}}$	L*er*	1.19	***	1.51	**
$\boldsymbol{hua2_{\mathrm{Col}}}$	Col-0	0.88	***	0.42	***

Legends of supplementary figures

Figure S1. The PHENOPSIS automated phenotyping platform. 504 plants are grown simultaneously under tightly-controlled conditions. (A): Top view of the PHENOPSIS growth chamber. (B) Lateral view of the PHENOPSIS growth chamber with the mobile weighing scale below the pots, and the mobile arm delivering water and holding camera above the pots.

Figure S2. Relationship between soil water content and soil water potential. Soil water potential was determined using a potentiometer (WP4-T dewpoint meter, Decagon Devices, Pullman, WA 99163, USA) during soil drying (from 0.35 to 0.06 g H_2O g^{-1} dry soil).

Figure S3 Heatmap of genetic (above-diagonal) and phenotypic (below-diagonal) correlations between traits in L*er* x Cvi RI lines under WD and HT. Plants were grown under control (CT, 20 °C) and high (HT, 30 °C) air temperatures and under well-watered (WW, 0.35 g H_20 g^{-1} dry soil) and water deficit (WD, 0.20 g H_20 g^{-1} dry soil) soil conditions. Age at reproduction (d), vegetative dry mass (mg), reproductive dry mass (mg), total leaf area (cm^2), LMA (g m^{-2}), RWC (%), stomatal density (st. mm^{-2}), mass- and area- based net photosynthetic rates (A_{mass} and A_{area} in nmol CO_2 g $^{-1}$ s^{-1} and nmol CO_2 cm^{-2} s^{-1}, respectively), mass- and area- based transpiration rates (T_{mass} and T_{area} in mg H_2O $mg^{-}1$ d^{-1} and mg H_2O cm^{-2} d^{-1}, respectively), and RGR (mg d^{-1} mg^{-1}). The coefficients of phenotypic correlation between traits were estimated as the Pearson's product moments. The coefficients of genetic correlation were estimated by dividing the covariance between RI line means for each pair of traits by the product of the square roots of among-line variance components for each trait. Colors vary from dark blue (higher negative values) to dark red (higher positive values). All traits were log-10 transformed. In each condition, n = 4 individuals x 120 RI lines.

Figure S4. Distribution of the 12 phenotypic traits in each environment. Curves represent the density of the distribution for the 12 phenotypic traits. Light blue: CTxWW; dark blue: CTxWD; orange: HTxWW; and dark red: HTxWD. Vegetative (A) and reproductive (C) dry masses (mg), age at reproduction (d) (B), total leaf area (cm^2) (D), leaf dry mass per area (LMA, g m^{-2}) (E), relative water content (RWC, %) (F), stomatal density (mm^{-2}) (G), mass-based net photosynthetic (H) and transpiration (J) rates (A_{mass}, nmol CO_2 s^{-1} g^{-1} and T_{mass}, mg H_2O d^{-1} mg^{-1}, respectively), area-based net

photosynthetic (I) and transpiration (K) rates (A_{area}, nmol CO_2 s^{-1} cm^{-2} and T_{area}, mg H_2O d^{-1} cm^{-2}, respectively), and relative growth rate (RGR, mg d^{-1} mg^{-1}) (L).

Figure S5. QTL analysis of 9 phenotypic traits within the four environments. (A), (B), (C) and (D): CTxWW, CTxWD, HTxWW, and HTxWD, respectively. From column 1 to 9: age at reproduction (days), vegetative dry mass (mg), reproductive dry mass (mg), total leaf area (cm^2), leaf mass per area (LMA, g m^{-2}), mass-based net photosynthetic rate (A_{mass}, nmol s^{-1} g^{-1}), mass-based transpiration rate (T_{mass}, mg d^{-1} g^{-1}), and absolute growth rate (mg d^{-1}). Arrows length represents confidence interval and arrows color represents the percent of variability explained by each QTL (< 5% to > 25%: lighter grey to black, respectively). Arrows direction represents the effect of Cvi alleles against L*er* alleles. Dashed lines represent significant epistatic interactions between QTL ($P < 0.01$).

Figure S6. Allelic effects of three QTL on the reactions norms under contrasted temperature and watering treatments. Trait values depending on the alleles (L*er* or Cvi) at *CRY2* (first column), *MSAT2.22* (second column) and *FD.98C* (third column). Plants were grown under control (CT, 20 °C) and high air temperature (HT, 30 °C) and under well-watered (WW, 0.35 g H_20 g^{-1} dry soil) and water deficit (WD, 0.20 g H_20 g^{-1} dry soil) conditions. Light blue: CTxWW; dark blue: CTxWD; orange: HTxWW; and dark red: HTxWD. Error bars represent 99.9%-confidence intervals. Significance levels of planned pairwise comparisons for allelic effect within each treatment following two-ways ANOVA: *** = $P < 0.001$; ** = $P < 0.01$; * = $P < 0.05$; ° = $P < 0.1$. n = 51 and 69 lines carrying L*er* alleles and Cvi alleles at *CRY2*, respectively. n = 69 and 51 lines carrying L*er* alleles and Cvi alleles at *MSAT2.22*, respectively. n = 66 and 54 lines carrying L*er* alleles and Cvi alleles at *FD.98C*, respectively.

Figure S7. Examples of leaf hyponastic movements observed in response to HT. (A) Rosette under CTxWW. (B) Rosette under HTxWW.

Figure S1.

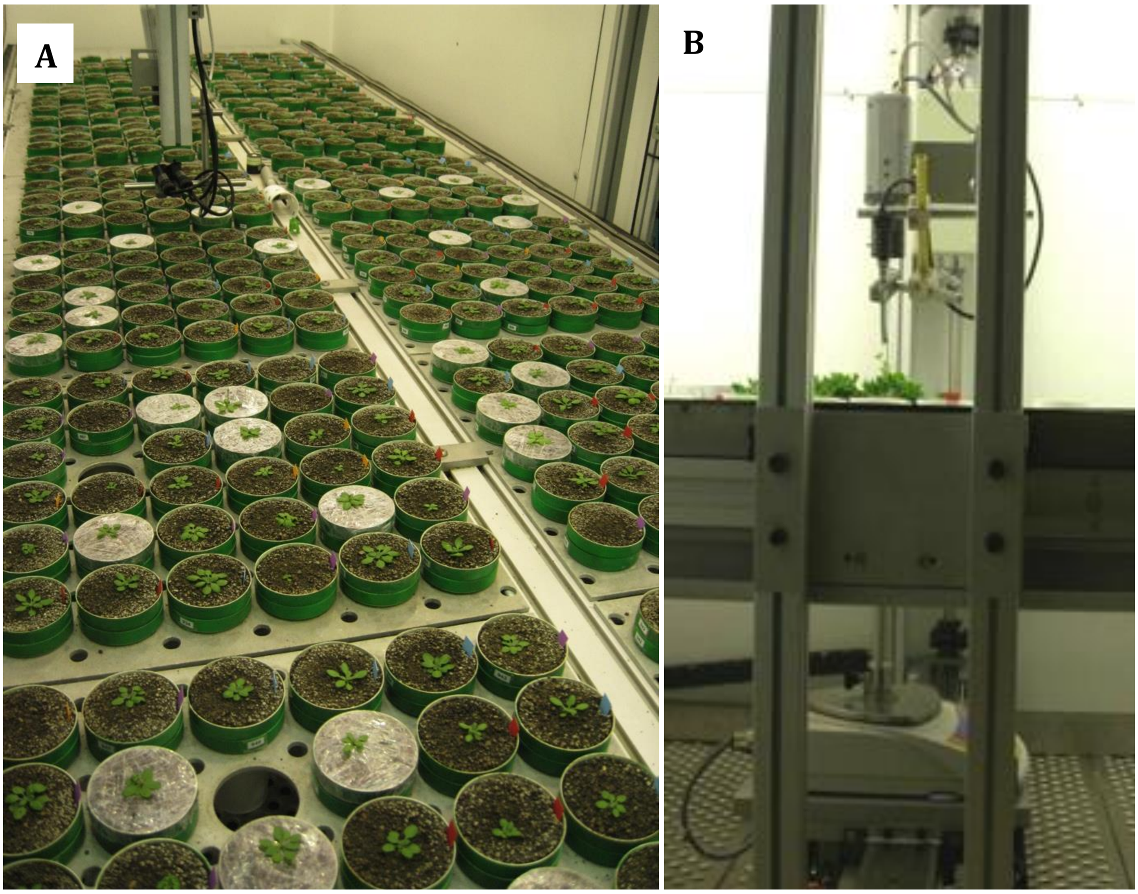

Figure S2.

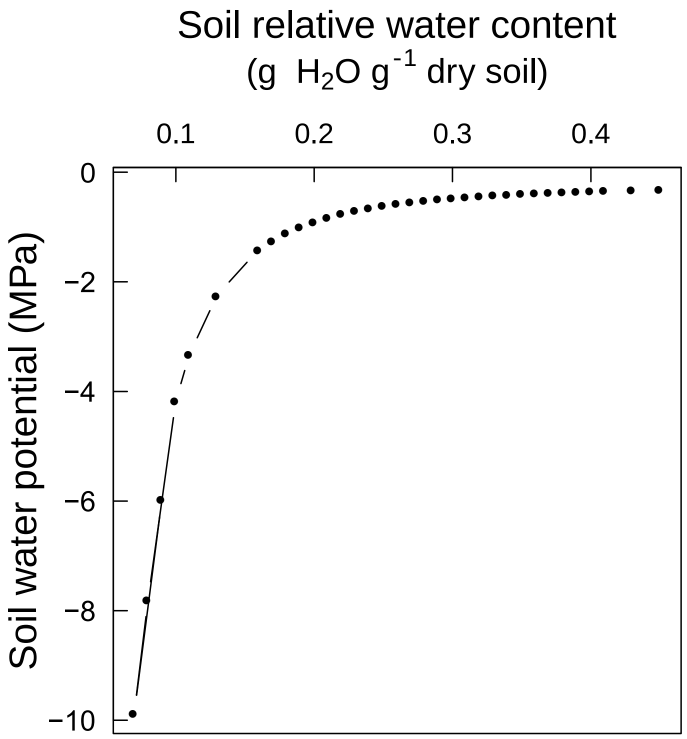

Soil relative water content
(g H2O g-1 dry soil)
0.1
0.2
0.3
0.4
Soil water potential (MPa)
0
−2
−4
−6
−8
−10

Figure S3.

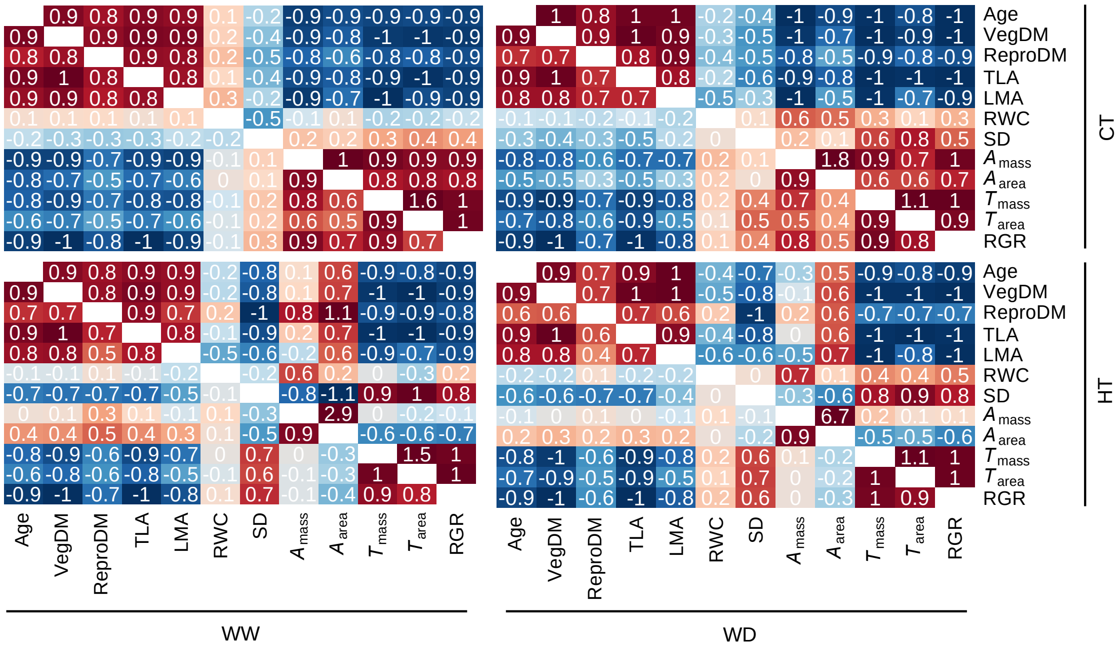
Age
VegDM
ReproDM
TLA
LMA
RWC
SD
A_{mass}
A_{area}
T_{mass}
T_{area}
RGR
CT
HT
WW
WD

Figure S4.

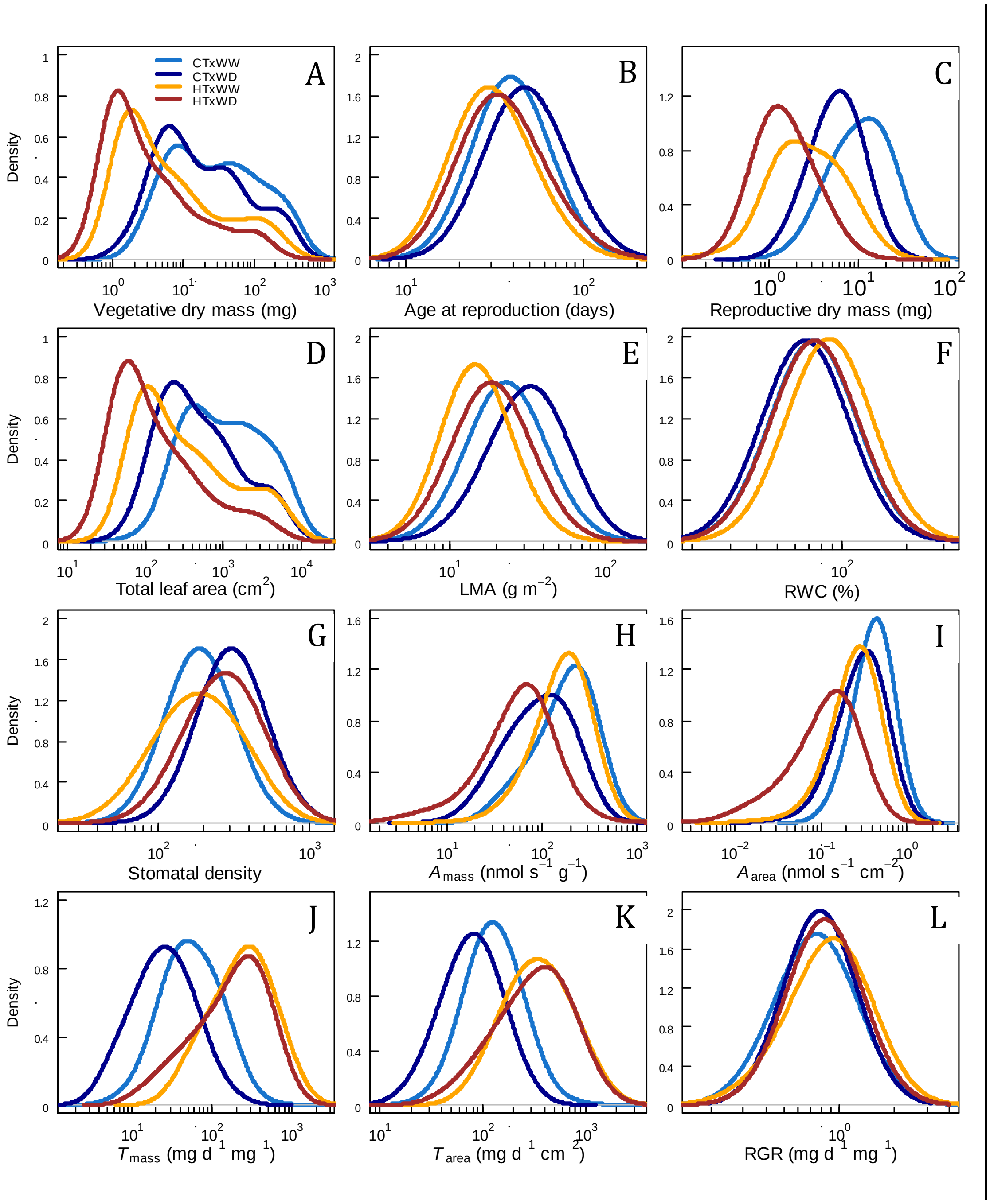

CTxWW
CTxWD
HTxWW
HTxWD
A
B
C
D
E
F
G
H
I
J
K
L
Density
Vegetative dry mass (mg)
Age at reproduction (days)
Reproductive dry mass (mg)
Total leaf area (cm2)
LMA (g m−2)
RWC (%)
Stomatal density
A mass (nmol s−1 g−1)
A area (nmol s−1 cm−2)
T mass (mg d−1 mg−1)
T area (mg d−1 cm−2)
RGR (mg d−1 mg−1)

Figure S5.

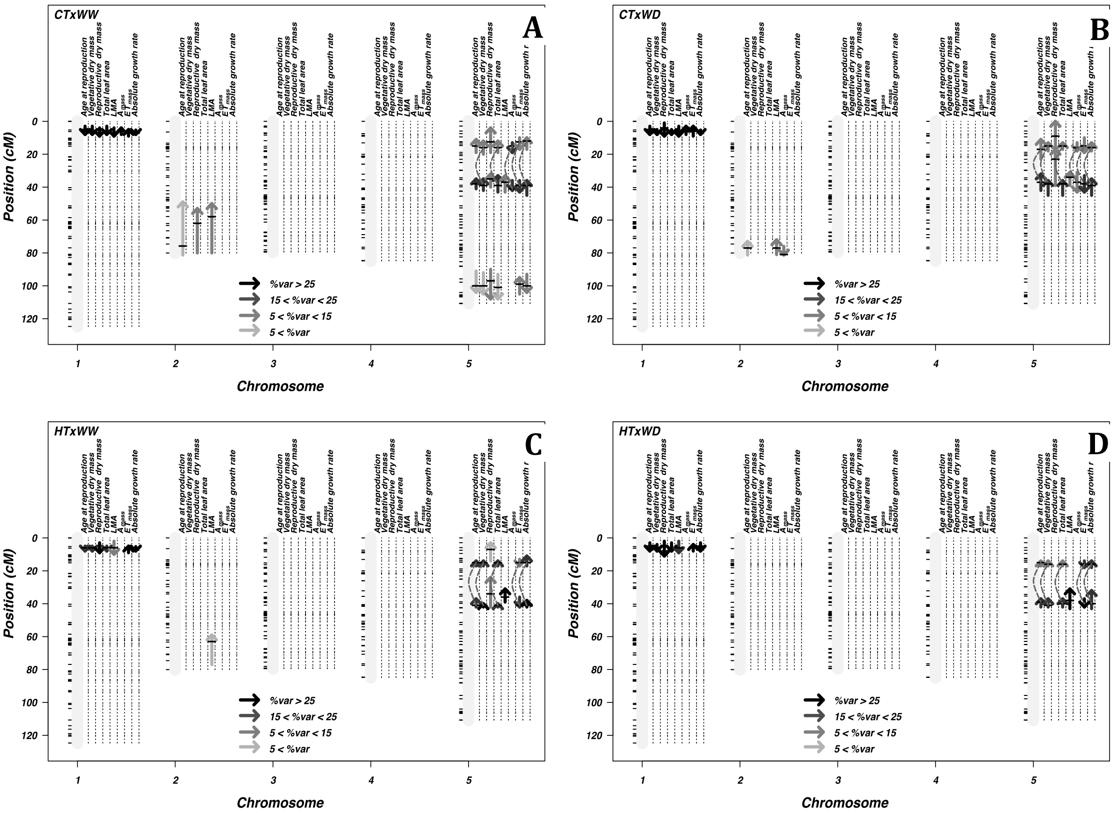
CTxWW
A
CTxWD
B
HTxWW
C
HTxWD
D
Age at reproduction
Vegetative dry mass
Reproductive dry mass
Total leaf area
LMA
Absolute growth rate
Position (cM)
Chromosome
%var > 25
15 < %var < 25
5 < %var < 15
5 < %var

Figure S6.

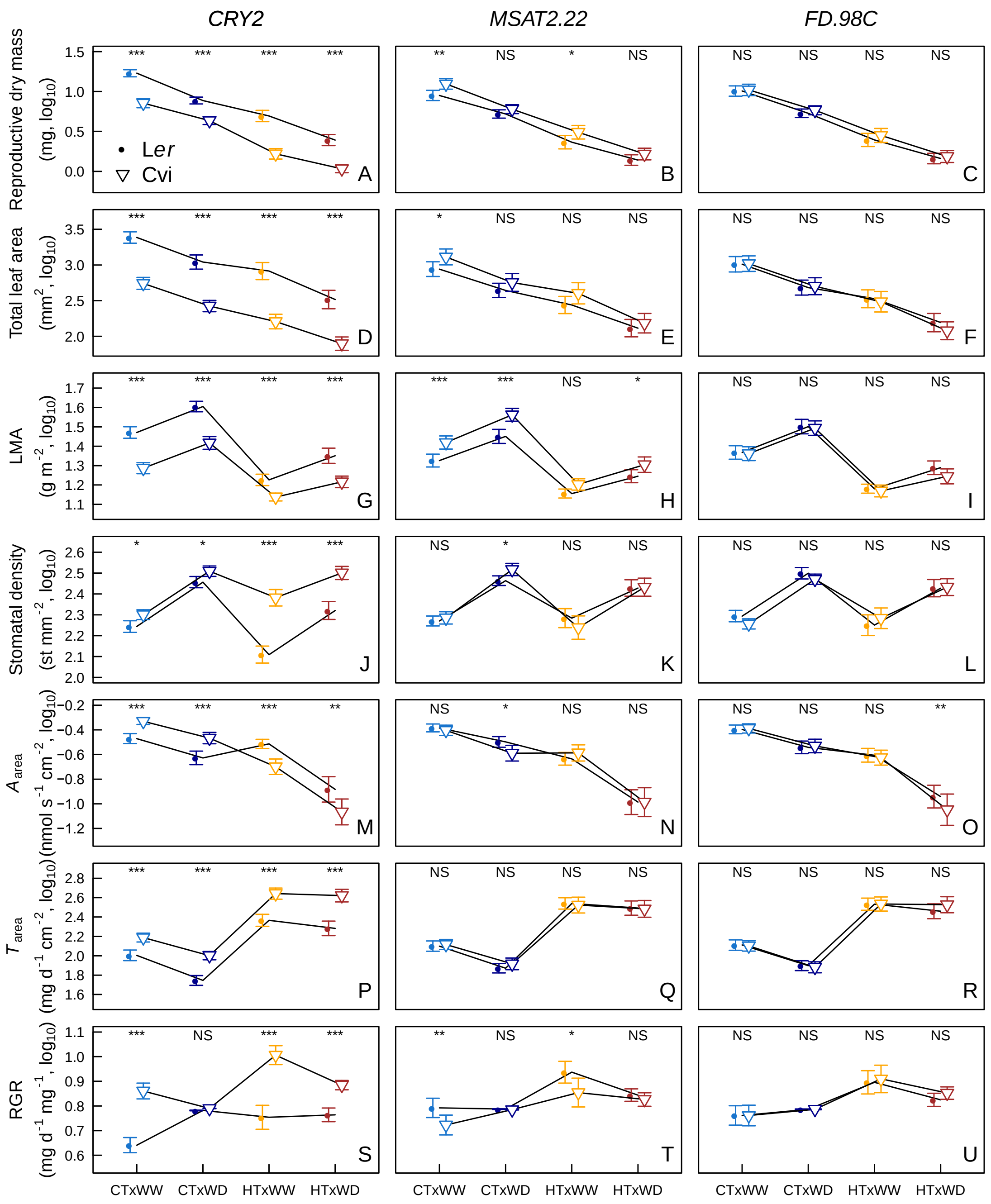
CRY2
MSAT2.22
FD.98C
Reproductive dry mass (mg, log10)
Total leaf area (mm2, log10)
LMA (g m-2, log10)
Stomatal density (st mm-2, log10)
Aarea (nmol s-1 cm-2, log10)
Tarea (mg d-1 cm-2, log10)
RGR (mg d-1 mg-1, log10)
Ler
Cvi
CTxWW
CTxWD
HTxWW
HTxWD
A
B
C
D
E
F
G
H
I
J
K
L
M
N
O
P
Q
R
S
T
U

Figure S7.

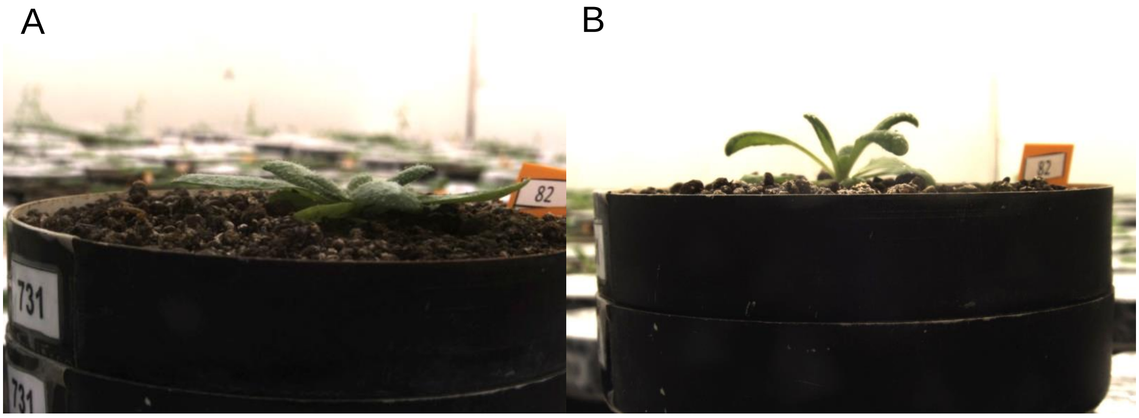

Supplement: Supplementary Data [file supp_eru364_jexbot123554_file001.pdf]
